# Genetic identification of source and likely vector of a widespread marine invader

**DOI:** 10.1002/ece3.3001

**Published:** 2017-05-11

**Authors:** Stacy A. Krueger‐Hadfield, Nicole M. Kollars, Allan E. Strand, James E. Byers, Sarah J. Shainker, Ryuta Terada, Thomas W. Greig, Mareike Hammann, David C. Murray, Florian Weinberger, Erik E. Sotka

**Affiliations:** ^1^ Department of BiologyUniversity of Alabama at BirminghamBirminghamALUSA; ^2^ Grice Marine Laboratory and the Department of BiologyCollege of CharlestonCharlestonSCUSA; ^3^ Odum School of EcologyUniversity of GeorgiaAthensGAUSA; ^4^ United Graduate School of Agricultural SciencesKagoshima UniversityKagoshima CityJapan; ^5^ NOAA/National Ocean ServiceCenter for Coastal Environmental Health and Biomolecular ResearchCharlestonSCUSA; ^6^ GEOMAR Helmholtz‐Zentrum für Ozeanforschung KielKielGermany; ^7^ Present address: Center for Population BiologyUniversity of CaliforniaDavisCAUSA

**Keywords:** algae, biological invasion, Northwest Pacific, oysters, phylogeography, population genetics

## Abstract

The identification of native sources and vectors of introduced species informs their ecological and evolutionary history and may guide policies that seek to prevent future introductions. Population genetics provides a powerful set of tools to identify origins and vectors. However, these tools can mislead when the native range is poorly sampled or few molecular markers are used. Here, we traced the introduction of the Asian seaweed *Gracilaria vermiculophylla* (Rhodophyta) into estuaries in coastal western North America, the eastern United States, Europe, and northwestern Africa by genotyping more than 2,500 thalli from 37 native and 53 non‐native sites at mitochondrial *cox*1 and 10 nuclear microsatellite loci. Overall, greater than 90% of introduced thalli had a genetic signature similar to thalli sampled from the coastline of northeastern Japan, strongly indicating this region served as the principal source of the invasion. Notably, northeastern Japan exported the vast majority of the oyster *Crassostrea gigas* during the 20th century. The preponderance of evidence suggests *G. vermiculophylla* may have been inadvertently introduced with *C. gigas* shipments and that northeastern Japan is a common source region for estuarine invaders. Each invaded coastline reflected a complex mix of direct introductions from Japan and secondary introductions from other invaded coastlines. The spread of *G. vermiculophylla* along each coastline was likely facilitated by aquaculture, fishing, and boating activities. Our ability to document a source region was enabled by a robust sampling of locations and loci that previous studies lacked and strong phylogeographic structure along native coastlines.

## 1 Introduction

Non‐native species represent one of the greatest threats to native biodiversity by homogenizing the Earth's biota and altering community processes and ecosystem function. Predicting the environmental and evolutionary processes that facilitate invasions is one of the major goals of invasion biology (Kolar & Lodge, 2001). For a species with a broad distribution and strong population genetic structure in its native range, the pooling of all native populations with which to compare against introduced populations may provide an uninformative contrast. Nonsource populations in the native range may not share the same opportunities for invasion because the vector(s) may be absent (Miler et al., 2002) or the necessary ecological or evolutionary characteristics that enable successful colonization are missing (Colautti & Lau, 2015; Estoup & Guillemaud, 2010). Identifying population source(s) and elucidating invasion pathways are fundamental to the investigation of subsequent evolution in recipient regions (Bock et al., 2015), the identification of other invaders from the same region or as a result of the same vector (Brawley et al., 2009), and the development of effective management strategies (Dlugosch & Parker, 2008). For most invasions, our knowledge about pathways and vectors is largely based on historical and observational data.

In the marine environment, maritime shipping and aquaculture have long been implicated in the introduction of non‐native species, particularly in estuarine habitats where these activities are more intense (Ruiz et al., 1997, 2000; Ruesink et al., 2005; Preisler et al., 2009). Ballast water and hull fouling have received a great deal of attention (see, e.g., Carlton & Geller, 1993) and are often cited as the most likely vectors for many marine invasions (Ruiz et al., 2000, Seebens, Schwartz, Schupp, & Blasius, 2016). Population genetic tools and advances in statistical techniques have greatly facilitated our understanding of invasion pathways, in part because these data allow independent tests of predictions that stem from observational data (Estoup & Guillemaud, 2010; Geller, Darling, & Carlton, 2010). For example, shipping from the Gulf of Mexico and the eastern seaboard of the United States was the likely vector in the introductions of the ctenophore *Mnemiopsis leidyi* to the Black, Caspian, North, Baltic, and Mediterranean seas (Reusch, Bolte, Sparwel, Moss, & Javidpour, 2010). Voisin et al. (2005) found maritime traffic promoted recurrent invasions of the kelp *Undaria pinnatifida* to Australasia from its native range in the Northwest Pacific. For most studies, however, two limitations hamper genetic identification of source and pathway: (1) the lack of robust sampling of native and non‐native populations and (2) the lack of appropriately variable population genetic tools (Estoup & Guillemaud, 2010; Geller et al., 2010).

The Japanese oyster *Crassostrea gigas* has been proposed to be one of the main vectors facilitating the hitchhiking of estuarine invaders worldwide (Ruesink et al., 2005). For example, Bonnot (1935) documented the occurrence of invasive mollusks with imported Japanese oyster shipments to North American estuaries. Despite the recognized importance of *C. gigas* exports as a vector of invasion, to our knowledge, only one study has attempted to identify the source populations by genotyping native and non‐native populations of estuarine invaders associated with oysters. Based on genotypic diversity in Japan and the western coast of North America, the invasion of the Asian mud snail *Batillaria attramentaria* as well as one of its trematode parasites originated in the main region of Japanese *C. gigas* exports in the Miyagi Prefecture (i.e., Matsushima or Mangoku Bays; Miura, Torchin, Kuris, Hechinger, & Chiba, 2006).

We explored the native and non‐native population genetic structure of the haploid–diploid seaweed *Gracilaria vermiculophylla* (Ohmi) Papenfuss (Rhodophyta), which has spread from its native distribution in the northwestern Pacific Ocean to virtually every high‐salinity, temperate estuary in Europe and North America (Byers, Gribben, Yeager, & Sotka, 2012; Saunders, 2009; Weinberger, Buchholz, Karez, & Wahl, 2008). *G. vermiculophylla* can profoundly transform estuarine ecosystems (Byers et al., 2012; Thomsen, Stæhr, Nejrup, & Schiel, 2013) and result in negative economic impacts (Freshwater et al., 2006). *G. vermiculophylla* was first identified through molecular barcoding from samples collected in 1979 in Baja California (Bellorin, Oliveira, & Oliveira, 2004), 1994 in Elkhorn Slough (Bellorin et al., 2004), 1998 in the Chesapeake Bay (Thomsen, Gurgel, & McGlathery, 2006), and 1995 in France (Rueness, 2005), but these dates are unlikely the first dates of establishment due to poor taxonomic resolution in the Gracilariales (Gurgel & Fredericq, 2004).

Despite a flurry of studies assessing the impacts of the *G. vermiculophylla* invasion (e.g., Hammann, Buchholz, Karez, & Weinberger, 2013; Nyberg & Wallentinus, 2009; Thomsen et al., 2013), the source of the invasion is known only at a coarse spatial scale. Kim, Weinberger, and Boo (2010) sequenced a portion of the mitochondrial locus cytochrome oxidase subunit one and found the non‐native range was dominated by a single haplotype (Haplotype 6), which was found in the Sea of Japan/East Sea. Unfortunately, their conclusions were limited by population sampling, as they did not sequence any thalli from the Pacific coastline of northeastern Japan (Honshu and Hokkaido Islands), areas of important aquaculture activities not only for *C. gigas* (Byers, 1999; Ruesink et al., 2005), but also agar extraction of *Gracilaria* spp., including *G. vermiculophylla* (Okazaki, 1971). The investigation of the underlying evolutionary processes facilitating this widespread invasion is limited due to the uncertainty of the invasion source(s).

In a previous study describing the impacts of the invasion on genetic diversity and the haploid–diploid life cycle, we genotyped greater than 2,000 thalli sampled from 30 native sites, with an emphasis on the Japanese archipelago (23 of the 30 sites), and 35 non‐native sites along the coastlines of western and eastern North America and Europe (Krueger‐Hadfield et al., 2016). There was an ecological shift from hard to soft substratum during the invasion of North American and European estuaries by *Gracilaria vermiculophylla* that resulted in a shift from sexual to asexual reproduction as hard substratum is a necessity for algal spore recruitment (Krueger‐Hadfield et al., 2016). Non‐native sites were dominated by diploid tetrasporophytes (>80% diploid thalli, on average) as a result of asexual fragmentation. We also found comparable levels of genetic diversity between native and non‐native sites, suggesting highly genetically diverse inocula, multiple invasions or both (Krueger‐Hadfield et al., 2016).

In order to investigate the global pathways and genetic structure of the *Gracilaria vermiculophylla* invasion, we analyzed the spatial genetic structure of the thalli previously genotyped (Krueger‐Hadfield et al., 2016) as well as thalli from 25 new native and non‐native sites. Our results pinpoint the Pacific shorelines of northeastern Japan as the ultimate source of introduced populations. Based on ecological, genetic, and historical evidence, we further suggest that *G. . vermiculophylla* hitchhiked with the *C. gigas* exports from Japan during the 20th century.

## 2 Materials and Methods

### 2.1 Data generation

#### 2.1.1 Sample collection

**Figure 1:**
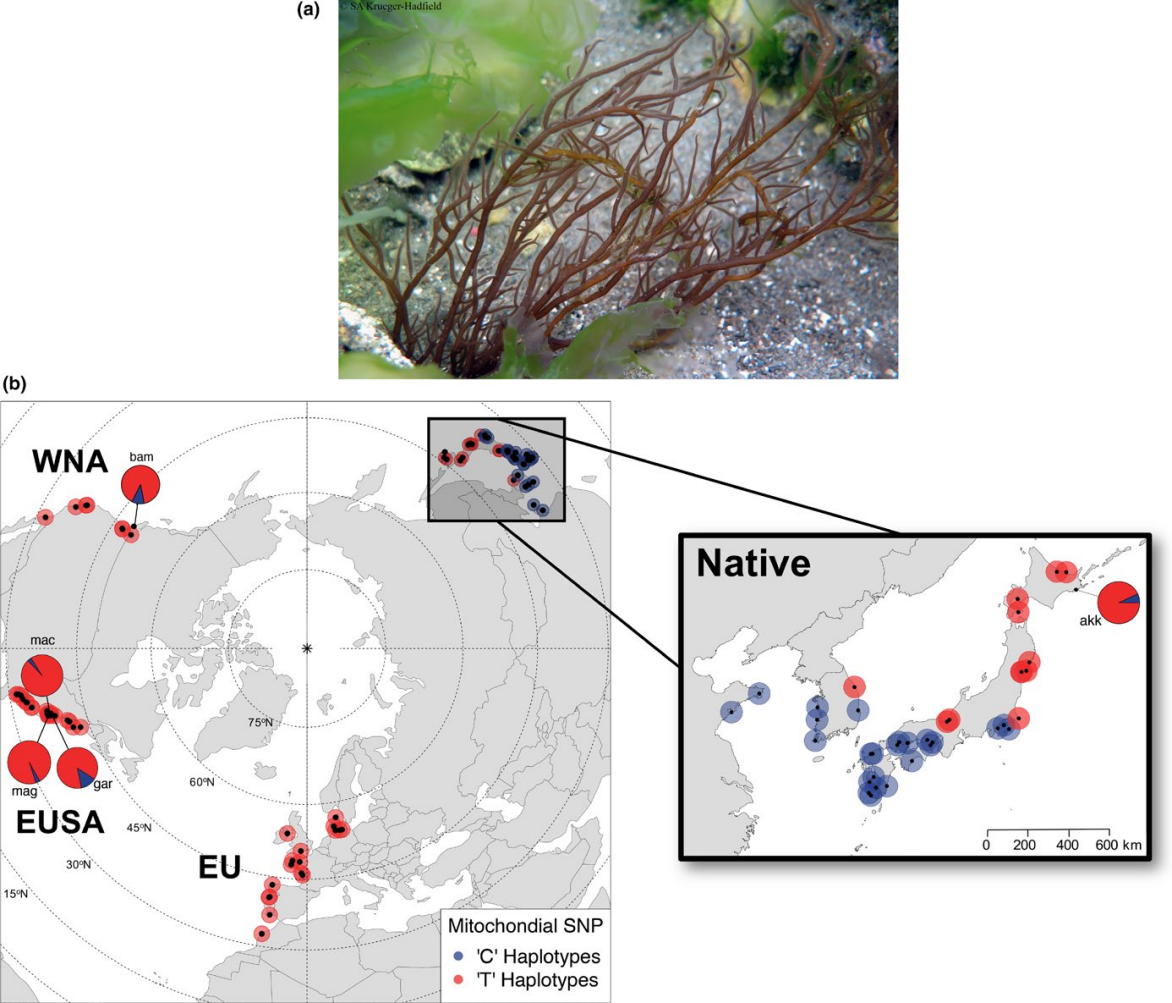
(a) *Gracilaria vermiculophylla* attached to hard substratum in a tide pool in the native range. Photographic credit: S.A. Krueger‐Hadfield. (b) Geographic distribution of the mitochondrial SNP frequency among sampled sites of *G. vermiculophylla* in the native range (Northwest Pacific) and non‐native range (WNA = western North America; EUSA = eastern United States; EU = British Isles, continental Europe, and northwestern Africa). At most sites, thalli were either “C” (blue) or “T” (red) at the 945th bp of the sequenced *cox*1 gene from Kim et al. (2010). At five sites, both “C” and “T” SNPs were detected and relative frequencies are shown by pie charts. Sampling information is provided in Table S1, and mitochondrial sequencing and genotyping data are provided in Figure S1 and Table S2

Algal thalli were sampled from 90 sites across the Northern Hemisphere from 2012 to 2016 (Table S1). Thirty‐seven sites were from the native range and sampled along the coastlines of China, South Korea, and Japan (Figure 1, Table S1, Figure S1). Fifty‐three sites were sampled along the coastlines of western North America (hereafter, WNA), the eastern United States (hereafter, EUSA), and northwestern Africa and Europe, including the British Isles (hereafter, EU).

The species identity of all thalli was confirmed either using DNA barcoding (Kim et al., 2010) or the amplification of 10 species‐specific microsatellite markers (Kollars et al., 2015; Krueger‐Hadfield et al., 2016). *Gracilaria vermiculophylla* thalli from native sites were attached via holdfasts to hard substrata (i.e., bedrock, pebbles, gastropod shells), whereas, in the non‐native range, there was a spectrum of sites ranging from fully attached to fully free‐floating *G. vermiculophylla* thalli (Krueger‐Hadfield et al., 2016; Krueger‐Hadfield & Sotka, unpublished data). At all sites, samples were collected with at least one meter separating each putative genet to avoid sampling the same genet twice (Guillemin et al., 2008; Krueger‐Hadfield et al., 2016). Ploidy was determined by observing reproductive material under a binocular microscope (40×) and/or using microsatellite loci (following Krueger‐Hadfield et al., 2016).

#### 2.1.2 DNA extraction, microsatellite and mitochondrial cox1 amplification and genotyping

Total genomic DNA was isolated and simplex PCRs of 10 microsatellite and fragment analysis were performed following Kollars et al. (2015) and Krueger‐Hadfield et al. (2016; see also Table S1). However, for sites sampled in 2015–2016 (Table S1), we only targeted and extracted phenotypically diploid thalli. In total, we genotyped 2,192 diploid and 785 haploid thalli across 90 native and non‐native sites. Only diploid thalli were used for subsequent microsatellite data analyses as the diploids dominated the non‐native range (Krueger‐Hadfield et al., 2016).

The mitochondrial gene *cox*1 was amplified using the primer sets 43F (Geraldino, Yang, & Boo, 2006) and 880R (Yang et al., 2008) and 622F (Yang et al., 2008) and 1549R (Geraldino et al., 2006) for 201 thalli sampled across both the native and introduced ranges (Table S2, Figure S1). We did not distinguish between haploid and diploid thalli for the mitochondrial sequencing. PCR amplification was performed on a total volume of 25 μl, containing 0.5 U of *taq* DNA polymerase, 2.5 mmol/L of each dNTP, 2 mmol/L MgCl_2_, 1× reaction buffer, 250 nmol/L of each primer, and 5 μl of DNA and PCR conditions previously described (Yang et al., 2008). Approximately, 5 μl of PCR product with 1 μl of Orange G loading dye was visualized on 1.5% agarose gels and 1 μl of ethidium bromide.

One microliter of ExoSAP‐It was added to 7 μl of PCR product (Affymetrix, Santa Clara, CA, USA) and incubated for 15 min at 37°C followed by 15 min at 80°C. Four microliters of 2 μmol/L primer was added to each product and sequenced in the forward direction commercially by Eurofins Genomics (Louisville, KY, USA).

We discovered a single nucleotide polymorphism (C/T) at the 945th base pair within the mitochondrial *cox*I fragment, which delineated among several haplotypes and could be genotyped using the restriction enzyme Af1III (New England Biolabs, Ipswich, MA, USA; ACGTG(T/C; Table 1, Table S2, Figure S1f). Restriction enzyme digestion was performed on thalli from each site using a total volume of 25 μl, containing 1× buffer, 10 U of AF1III and 5 μl of the *cox*1 PCR product amplified using the primers 622F (Yang et al., 2008) and 1549R (Geraldino et al., 2006) and under the following reaction conditions: 37°C for 2 h, 80°C for 20 min, and 20°C for 5 min. Restriction digest products were visualized on 1.5% agarose gels with 1 μl of ethidium bromide (Figure S1f).

**Table 1:** Haplotypic diversity across the native and non‐native ranges of *Gracilaria vermiculophylla*. a) Sites south and north of approximately 35°N in China, South Korea, and Japan were delineated based on C:T frequencies (see also Figure 1). We used the haplotype numbers assigned by Kim et al. (2010) as these covered the ~1,200 bp of *cox*1. We have extended Kim et al. (2010) and generated haplotype numbers for six new haplotypes (haplotypes 26–31; see Table S2). The proportion of the “C” and “T” SNPs are given for each region (see Table S1 for sampling sizes are each site). b) The biogeographic province (Briggs & Bowen, 2011) and ecoregions (Spalding et al., 2007) in which each of the native range haplotypes were found

(a) Region	“C” Haplotypes	“T” Haplotypes	C:T Ratio
South of ~35°N	1–4, 8–15, 17, 27, 30–31	None	1.00: 0.00
North of ~35°N	26	5–7, 16, 18, 28	0.01: 0.99
WNA	15	6, 19, 29	0.02: 0.98
EUSA	15	6	0.01: 0.99
EU	None	6, 18	0.00: 1.00

(b) Biogeographic province	Ecoregion	“C” Haplotypes	“T” Haplotypes
Cold temperate—Kurile	Sea of Okhotsk	None	6
	Oyashio Current	26	6
	Northeastern Honshu	None	6
Cold temperate—Oriental	Sea of Japan	None	5–7
	Northeastern Honshu	None	6, 18, 28
	Yellow Sea	1–2, 17	None
Warm temperate—Sino‐Japanese	Sea of Japan	4	None
	East China Sea	2–4; 8–13; 30–31	None
	Central Kuroshio Current	14–15; 27	16

### 2.2 Data Analyses

#### 2.2.1 Mitochondrial analyses

Sequences were edited using *4Peaks* (Nucleobytes, The Netherlands), aligned with the haplotypes from Kim et al. (2010) using *Muscle* (Edgar, 2004) in *Seaview* ver. 4.6 (Gouy, Guindon, & Gascuel, 2010) with default parameters. We assigned haplotype numbers using Kim et al. (2010), which sequenced a ~1200‐base pair fragment. We also defined six new ~1200‐bp haplotypes that were not previously sequenced (Kim et al., 2010) using *DnaSP*, ver. 5.10.1 (Librado & Rozas, 2009). We ignored the haplotype designations of Gulbransen, McGlathery, Marklund, Norris, & Gurgel (2012) because their haplotypes covered only a subset of this larger ~1200‐bp fragment.

A phylogeny of the haplotypes from Kim et al. (2010) and new haplotypes uncovered in this study was constructed using the Hasegawa–Kishino–Yano model (HKY) plus gamma (Hasegawa, Kishino, & Yano, 1985), one of the appropriate models detected using ModelTest (Darriba, Taboada, Doallo, & Posada, 2012; Guindon & Gascuel, 2003), as implemented in MEGA6 (Tamura et al. 2013). The resulting trees were edited using *FigTree* ver. 1.4.2 (http://tree.bio.ed.ac.uk/software/figtree/).

#### 2.2.2 Ploidy and genet determination

The number of repeated identical multilocus microsatellite genotypes (MLGs) was computed using *GenClone* ver. 2.0 (Arnaud‐Haond & Belkhir, 2006) and a custom *R,* ver. 3.2.2 (R Core Team, 2016) routine following (Arnaud‐Haond, Duarte, Alberto, & Serrao, 2007; Parks & Werth, 1993), now implemented in *poppr* ver. 2.2.1 (Kamvar, Brooks, & Grünwald, 2015; Kamvar, Tabima, & Grünwald, 2014). *P*_sex_, or the probability for a given MLG to be observed in *N* samples as a consequence of different sexual reproductive events, was calculated for each repeated MLG. If *P*_sex_ was greater than 0.05, duplicated multilocus genotypes were considered as different genets. If *P*_sex_ was smaller than 0.05, the duplicated MLGs were considered as ramets (or clones) of the same genet.

Studies on the population genetics of clonal organisms remove repeated MLGs before calculating heterozygosity and other *F*‐statistics in order to avoid distorting these estimates (e.g., Halkett et al., 2005; Krueger‐Hadfield, Collen, Daguin‐Thiébaut, & Valero, 2011; Sunnucks, de Barro, Lushai, Maclean, & Hales, 1997). Following this convention, we chose the conservative approach of performing the following analyses for diploid MLGs for which *P*_sex_ > 0.05 (i.e., one thallus per genotype based on *P*_sex_).

#### 2.2.3 Within and between population genetic variation

Kollars et al. (2015) and Krueger‐Hadfield et al. (2016) provided locus information, the number of alleles, and basic summary statistics. For each site, the average expected heterozygosity (*H*_*E*_) was calculated using *GenAlEx*, ver. 6.5 (Peakall & Smouse, 2006, 2012). An estimate of the mean expected number of alleles (*A*_*E*_) was computed using rarefaction implemented in the program *HP‐Rare,* ver. 1.0 (Kalinowski, 2005) on the smallest sample size of 10 diploids (i.e., 20 alleles). Finally, the number of expected MLGs (*eMLGs*) at the smallest sample size (*n* = 10 diploid thalli) was estimated using rarefaction in the *R* package *poppr,* ver. 2.2.1 (Kamvar et al., 2014, 2015). For the latter two diversity metrics, sites with less than 10 diploid thalli were excluded from calculations.

We calculated pairwise genetic differentiation based on allele identity (*F*_ST_) and allele size (ρ_ST_) among sites along each of four coastlines: Japan, WNA, EUSA, and EU using *genepop* ver. 4.4 (Rousset, 2008). We measured geographic distance (km) following the contours of each coastlines between all site pairs using the measure distance function in Google^®^ Maps. We performed Mantel tests in order to detect relationships between the genetic and geographic distance along each of the four coastlines using *R* (R Core Team, 2016).

#### 2.2.4 Multivariate analyses of microsatellite data

To assess relationships among multilocus genotypes, we pursued a discriminant analysis of principal components (DAPC), a multivariate analysis that avoids making strong assumptions about the underlying genetic model (Jombart, Pontier, & Dufour, 2009). DAPC finds the principal components that best summarize the differences among clusters that are found, while also minimizing within‐cluster variation (Jombart, Devillard, & Balloux, 2010), and is implemented in the *R* package *adegenet,* ver. 2.0.1 (Jombart, 2008; Jombart & Ahmed, 2011). The procedure first generates a principal component analysis (PCA) on predefined groups (see below). These PCs were, then, used as variables for a discriminant analysis that maximizes the intergroup component of variation.

We performed the DAPC with increasing numbers of PCs on 90% of our data, and then the remaining 10% of the individuals were projected onto the discriminant axes constructed by the DAPC. It was possible to measure how accurately the remaining 10% of the individuals were placed in multidimensional space (i.e., how well their position corresponds to their group membership). Based on this cross‐validation with the *xvalDapc* function, we retained 88 principal components (PCs) that explained 87.8% of the total variance for subsequent DAPCs (Figure S2a).

We estimated how well‐supported the group membership was relative to five a priori subregions using the *compoplot* function in *adegenet*. Posterior group memberships were utilized in order to indicate admixture or the misclassification when prior groups are used to conduct the DAPC. Based on the mitochondrial SNP, we divided the native range into the “C” haplotype group and the “T” haplotype group corresponding to the break between ~35°N in Japan and South Korea in which the two SNPs dominated to the south and north, respectively (Figure 1, Table 1). For the introduced range, we treated each coastline as a different group: WNA, EUSA, and EU. The stability of a priori group membership probabilities, derived from proportions of successful reassignments based on retained discriminant functions of DAPC, was high (>92%) for the native “C” haplotype and EUSA and EU subregions, but lower for the native “T” haplotype and WNA subregions (85.3% and 76.4%, respectively; Figure S2). Based on these reassignment frequencies, we performed the DAPC with the aforementioned five subregions.

Next, we predicted the assignment of thalli from one region to another using supplementary individuals that were not used in model construction as implemented in *adegenet*. We performed this analysis with training data composed of (1) only the native sites and (2) sites from the native range and two of the three introduced coastlines. We transformed “new data” using the centering and scaling of training data (i.e., (1) the native range alone or (2) the native range and the two introduced coastlines). Using the same discriminant coefficients, we predicted the position of the new individuals (i.e., (1) all introduced sites and (2) the one introduced coastline not used in the training data).

Because both native and non‐native populations of *Gracilaria vermiculophylla* are out of Hardy–Weinberg equilibrium (Krueger‐Hadfield et al., 2016), we chose to use Bayesian model‐based clustering that relaxes the HWE assumption as implemented in the software *instruct* (Gao, Williamson, & Bustamante, 2007). Simulations were performed using *instruct* with a model including both biparental inbreeding and admixture, where each individual drew some fraction of its genome from each of the *K* populations. A burn‐in of 300,000 repetitions and a run length of 500,000 were used for *K *=* *2 to *K *=* *30, where 20 chains were run for each *K*.

To evaluate the values of *K*, we analyzed *K *=* *2 to 30 clusters using *clumpak* (Kopelman, Mayzel, Jakobsson, Rosenberg, & Mayrose, 2015). *clumpak* identifies sets of highly similar runs across the 20 independent runs of each *K* generated with *instruct* and separates them into distinct major and minor modes. *clumpak* utilizes the software *clumpp* (Jakobsson & Rosenberg, 2007) in order to generate a consensus solution for each distinct mode using a Markov clustering algorithm that relies on a similarity matrix between replicate runs. Next, *clumpak* identifies an optimal alignment of inferred clusters and matches the clusters across the values of *K* tested.

We determined the optimal number of *K* using outputs from *instruct* and *clumpak*. First, we plotted the number of clusters against the values of DIC (± *SE*) and chose the value of *K* at the point at which the curve reached an asymptote. The lower the DIC value, the better fit of the model used (i.e., the number of *K*). Second, we used the number of independent chains out of 20 that generated the major mode and the highest mean similarity score from *clumpak*.

We constructed a neighbor‐joining (NJ) tree based on Jaccard distance using the *R* packages *ape* ver. 3.5 (Paradis, Claude, & Strimmer, 2004). The resulting trees were edited using *FigTree*.

## 3 Results

### 3.1 Mitochondrial structure across native and introduced ranges

Across 201 thalli sequenced from the native and non‐native ranges, we identified 10 distinct *cox*1 haplotypes (Figure S1, Table S2), four of which were described previously by Kim et al. (2010). Three groups of haplotypes were identified with a maximum‐likelihood (ML) analysis based on 19 previously described haplotypes (Kim et al., 2010) and the six new haplotypes from this study. The three clades were heterogeneously distributed across the native range (Figure S1a). Two clades were found south of ~35°N latitude: One consisted of 10 haplotypes including haplotypes 1, 2, 3, 9, 10, 11, 12, 17, 26, 30, 31 and the other clade of six haplotypes included 4, 8, 13, 14, 15, and 27 (Table 1, Figure S1a). The third clade of six haplotypes were only found sequenced north of ~35°N in the native range (Figure 1, Table 1, Figure S1). Seventy‐two of 77 thalli, or 94% of sequenced thalli, collected north of 35°N were haplotype 6, the haplotype that is known to dominate the introduced range (Kim et al., 2010).

Two single nucleotide polymorphisms, or SNPs (945th bp and 1040th bp from Kim et al., 2010; Table S2) delineated all haplotypes that occur north (“T”) from those that occurred south (“C”) of ~35°N in the native range (Figure S1, Table S2). Based on RFLP genotyping of 691 thalli collected south of ~35°N latitude, we found that all were “C” haplotypes (Figure 1, Table 1). An RFLP analysis of 375 thalli collected north of ~35°N revealed all were “T” haplotypes. The exceptions were three thalli from Akkeshi (akk) that belonged to the new “C” haplotype 26 that diverges from all other haplotypes by at least five base pairs (Figure S1a, Table S2).

In the non‐native range, 99.5% (1,597 of 1,605 thalli) of the thalli were “T” haplotypes and were dominated by haplotype 6 (82 of 101, or 81%, sequenced thalli; Figure 1, Table 1, Figure S1b‐d, Table S2). Only nine non‐native thalli were “C” haplotypes (three from Bamfield/bam, four from Gargatha/gar, one from Machipongo/mac, and one from Magotha/mag) and all belonged to haplotype 15. Haplotype 15 was originally sampled at Funabashi in Tokyo Bay (Kim et al., 2010), near the “C/T” haplotype break. The “T” haplotypes 19 (Elkhorn Slough/elk; Kim et al., 2010) and 29 (Bamfield/bam; this study) were detected in the non‐native range, but not in the native range, likely because of under‐sampling in northern Japan. For example, the “T” haplotype 18 was previously only sampled in France (Kim et al., 2010), but was found after sequencing thalli from Mangoku‐ura (mng; Figure S1, Table S2).

Taken together, mitochondrial data suggest the region north of ~35°N latitude was the source of the widespread Northern Hemisphere invasion (Figure 1). However, we could not resolve the sites that contributed to the invasion at finer spatial scales due to the lack of mitochondrial polymorphism within this northern native region.

### 3.2 Microsatellite structure across native and non‐native ranges

The ten microsatellite loci revealed profound genetic structure within the native range and identified cryptic genetic structure among and within non‐native coastlines that could not be detected with mitochondrial sequencing alone. We focus on each of these patterns in turn.

**Figure 2:**
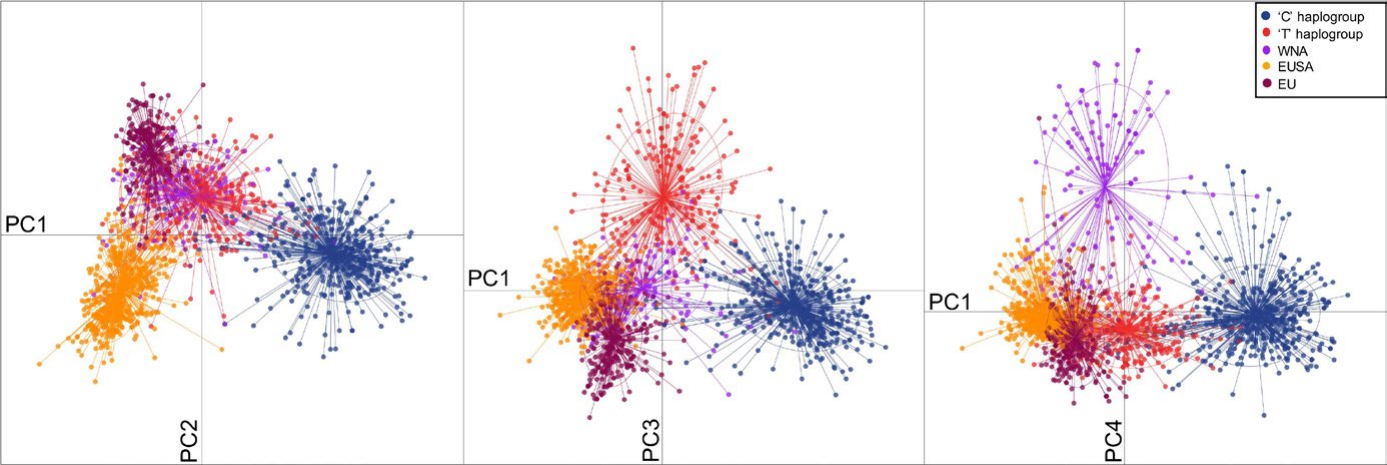
DAPC (discriminant analysis of principal components) relationships among microsatellite genotypes of *Gracilaria vermiculophylla*. We color‐coded individuals corresponding to five a priori groups with high reassignment frequencies (see Figure S2) as the “C” haplogroup (native sites dominated by the “C” mtSNP and south of ~35°N), the “T” haplogroup (native sites dominated by the “T” mtSNP and north of ~35°N), WNA (western North America), EUSA (eastern United States of America), and EU (Europe and northern Africa). The first four principal components are shown (PC1: 55.4%, PC2: 19.4%, PC3: 14.3%, PC4: 10.9%)

**Figure 3:**
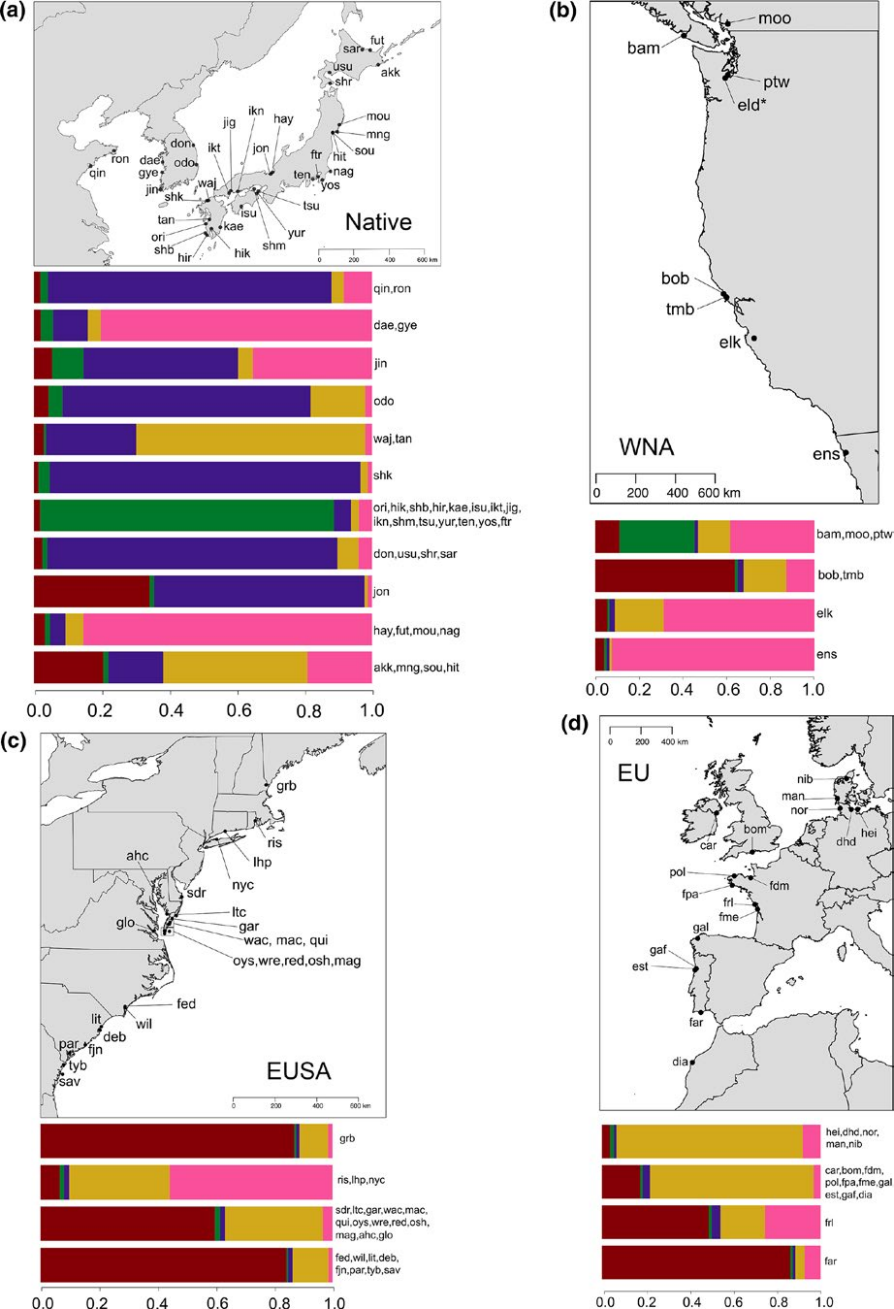
The mean assignment to five genetic clusters (colored as red, green, purple, gold, and pink) as generated using *instruct* (Gao et al., 2007) and *clumpak* (Kopelman et al., 2015). We grouped thalli across sites of similar genetic composition using a visual inspection of individual cluster assignment (see Figure S3e) and used barplots to display individual assignments averaged across sites for that group. As Eld Inlet (eld) had only one unique MLG based on *P*_sex_, it was excluded from Bayesian analyses, but is shown on the map

Within the native range, microsatellite genotypes distinguished northern sites dominated by “T” *cox*1 haplotypes and southern sites dominated by “C” haplotypes. This separation was evident along the first axis of a multivariate DAPC, which itself explained 55% of overall variation (Figure 2). “T” versus “C” differentiation was also confirmed by Bayesian clustering analyses in which sites were placed into subregions corresponding to genetic similarity (Figure 3, Figure S3, Table S3). At *K *=* *5, the optimal *K* based on the similarity score using *clumpak* and the curve of DIC estimates (Figure S3) showed strong differentiation within and between the “T” and “C” regions in the native range (see Figure S3b–f for individual‐level analyses and across *K*'s).

At finer geographic scales, there was a high degree of genetic differentiation among sites that were close in proximity (i.e., less than 100 km) in the native range. Genetic differentiation in the native range, as measured using allele identity (*F*_ST_) and allele size (ρ_ST_), was high on average (*~*0.47, Figure S4, Table S4). Genetic isolation was positively correlated with geographic distance when measured using allele identity (*F*_ST_), but not with allele size (ρ_ST_, Figure S4, Table S4).

In the non‐native range, the three continental shorelines were genetically differentiated and, thus, likely reflect different invasion histories. The DAPC analyses showed a separation of eastern United States (labeled as EUSA) thalli from the northern Japanese sites and other introduced shorelines along the second principal component axis, which itself explained ~19% of the variation (Figure 2). The British Isles, European and northwestern African (labeled as EU), and western North American (labeled as WNA) thalli were differentiated from other regions along the third (14.3% of variation) and fourth (10.9% of variation) principal component axes, respectively (Figure 2). With increasing numbers of genetic clusters using Bayesian analyses, introduced coastlines showed greater levels of genetic divergence from each other. At *K *=* *3, WNA sites were differentiated from the majority of EUSA and EU sites (Figure S3c), while at *K *=* *5, the EUSA and EU were genetically distinguishable (Figure S3e).

The strength of differentiation among populations along a shoreline differed across non‐native coastlines. Within WNA and EU shorelines, sites exhibited greater levels of genetic differentiation (average *F*_ST_ *~* 0.33 and ρ_ST_ ~ 0.20; Table S4) than was seen along the EUSA (average *F*_ST_ *~* 0.15 and ρ_ST_ ~ 0.18; Table S4). Significant patterns of isolation by distance (IBD) were detected along WNA and EU coastlines, but IBD was only marginally significant along the EUSA when measuring differentiation by allele identity (*F*_ST_; Figure S4, Table S4). In contrast, there were positive relationships between genetic differentiation and geographic distance along the EU and EUSA when measuring differentiation by allele size, but they were only marginally significant (ρ_ST_, Figure S4, Table S4).

It is likely that among‐site differentiation within the non‐native range reflected complex genetic origins that differ between coastlines. Along the WNA coastline, the Pacific Northwest (bam, pmo, and ptw), Californian (bob and tmb), Elkhorn Slough (elk), and Ensenada (ens) sites were composed of different genetic clusters (Figure 3; Figure S3). Similarly, a neighbor‐joining clustering analysis of site‐level genetic distances indicated that the WNA coastline has been separately invaded on two or three occasions, given that sites are in separate locations across the tree (Figure S5). However, the bootstrap support for the NJ tree was poor and, as a result, this analysis should be interpreted with caution.

Along the EUSA coastline, most sites were composed of a homogenous set of genetic constituents, regardless of the number of genetic clusters (Figure 3, Figure S3). The exceptions were the rocky‐shore sites of Long Island Sound (lhp and nyc) and Narragansett Bay (ris), which seemed to have genetic constituents more closely aligned with WNA and EU sites (Figure 3, Figure S3). In the NJ clustering analysis, the majority of the EUSA sites were also part of the same clade, with the exception of these three northeastern sites (ris, lhp, and nyc; Figure S5).

Along the EU coastline, Bayesian clustering revealed differences between sites sampled in Germany and Denmark (hei, dhd, nib, man, nor) versus a group of French (frl) and Portuguese populations (far), while the remaining populations in Ireland, the United Kingdom, France, Spain, Portugal, and Morocco reflected admixture and a similar set of genetic constituents (Figure 3, Figure S3). The NJ tree suggested a largely similar origin of all European populations, with the exception of the site in Dorset (bom) in the United Kingdom which clustered with a site in the Chesapeake Bay (ahc; Figure S5).

**Figure 4:**
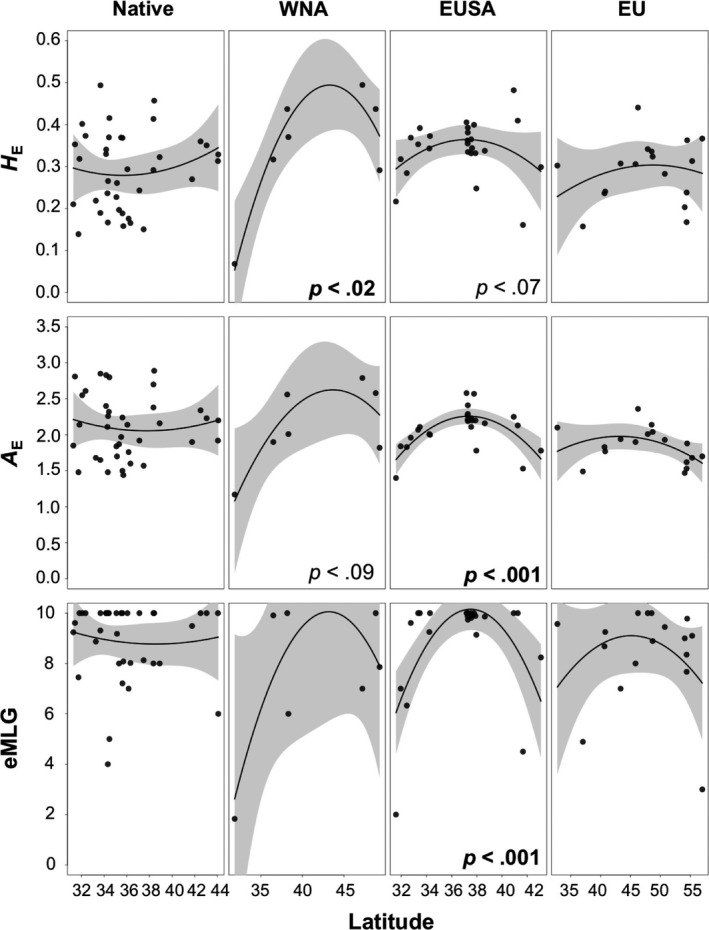
Genetic (*H*_*E*_ and *A*_*E*_) and genotypic (*eMLG*) diversity as a function of latitude along the coastlines of the native range, WNA, EUSA, and EU. Significance values are shown in the plots and bold if less than *p *=* *.05

There was a significant relationship between different measures of genetic and genotypic diversity (*H*_*E*_ *, A*_*E*_, and *eMLG*) versus latitude along the EUSA and for genetic diversity metrics (*H*_*E*_ and *A*_*E*_) versus latitude (Figure 4, Table S5). Highest diversity was found in the mid‐latitudes along each coastline. Similar patterns were found along the EU coastline, but none of the patterns were significant (Table S5).

### 3.3 Population sources for primary and secondary introductions

Several analyses of microsatellite data indicated the Pacific shoreline of northeastern Japan was the ultimate genetic source of introduced *Gracilaria vermiculophylla* thalli throughout the Northern Hemisphere. Blind assignment of introduced populations onto multivariate DAPC space created by genotypes from native sites suggested most non‐native thalli originated in three northern Japanese sites (791 thalli; ~83%): Soukanzan (sou), Mangoku‐ura (mng), and Akkeshi (akk, Figure 1, Figure S6). Of the remaining 164 thalli, 101 (~11%) were assigned to other northern “T” haplotype sites, principally along northeastern Honshu and eastern Hokkaido Islands (Figure 1, Figure S6). Of the remaining 63 thalli, 14 WNA thalli (~2%) were assigned to Yoshio (yos), a site on the Chiba Peninsula at the “C/T” break, and 15 EU thalli (~2%) and 22 EUSA thalli (~2%) were assigned to Shikanoshima (shk), located in northern Kyushu Island (Figure 1, Figure S6).

Both Bayesian (Figure S3) and neighbor‐joining clustering methods (Figure S5) supported the notion that non‐native coastlines were most closely aligned with the Pacific shoreline of northeastern Japan. With increasing *K*'s, the genetic clusters found in the northern “T” haplotype sites were the same clusters found predominately along each non‐native coastline (Figure 3, Figure S3). Similarly, NJ analyses also confirmed genetic similarity of Soukanzan (sou), Mangoku‐ura (mng), and Akkeshi (akk) with non‐native sites (Figure S5).

Finally, as briefly mentioned above, we found some evidence consistent with secondary introductions (i.e., movement of introduced thalli to other locations within the introduced range). The strongest evidence of secondary introduction came from the three sites from Long Island Sound and Narragansett Bay (nyc, lhp, ris). Bayesian clustering at *K *=* *5 (Figure 3, Figure S3) and the NJ analyses (Figure S5) suggested these sites were more closely aligned with WNA sites (elk, ens) than with other EUSA sites. In addition, blind assignment of non‐native populations onto multivariate DAPC space created by genotypes from sites in the native range and two of the three non‐native coastlines suggested non‐native thalli may have more complex primary and secondary invasions (Figure S6). For example, EUSA and EU thalli were assigned to sites from the central Californian coast in addition to the northeastern coastline of Japan.

## 4 Discussion

Using a combination of mitochondrial and microsatellite genotyping, we discovered profound phylogeographic structure in the native range of *Gracilaria vermiculophylla*, reflecting low levels of contemporary gene flow. Introduced populations of North America and Europe exhibited mitochondrial and nuclear genotypes largely indistinguishable to those of the Pacific coastline of northeastern Japan (i.e., northern Honshu and southeastern Hokkaido Islands). Because this area historically served as the principal source for oyster exports worldwide, we suggest that oysters were the key vector for the *G. vermiculophylla* invasion. We discuss each of these findings in detail below.

### 4.1 Phylogeographic structure in the native range

In comparison with well‐studied biogeographic provinces, such as those in northeast (NE) Atlantic, less is known about the evolutionary history of the organisms that inhabit the northwest (NW) Pacific and it is not clear whether hypotheses drawn from patterns in other regions apply (Cheang et al., 2010). Phylogeographic studies in the NW Pacific have been biased toward commercially exploitable fish and mollusks and the majority have been underappreciated within English‐language journals because of language barriers (Ni, Li, Kong, & Yu, 2014). The existing studies reveal that phylogeographic patterns complex due to intricate topography and dynamic current systems (Wang, 1999).

The marginal seas of the South China Sea, the East China Sea, and the Sea of Japan/East Sea may have served as glacial refugia, although intraspecific variation in demographic histories is common across taxa (Ni et al., 2014). He, Mukai, Chung, and Zhang (2015) found the amphibious mudskipper *Periophthalmus modestus* colonized northward from the South China Sea through the East China Sea along the coasts of Korea and Japan. Other studies have found higher haplotypic diversity in southern South Korea and southern Japan (i.e., East China Sea) as compared to Honshu and Hokkaido populations (Ho, Kwan, Kim, & Won, 2014; Kim, Hoarau, & Boo, 2012), suggesting northward colonization. However, although studies have found southern clade haplotypes decrease to the north (Azuma & Chiba, 2015), no studies, to our knowledge, have found as sharp a break as observed in our study of *Gracilaria vermiculophylla* in the NW Pacific (Figure 1, Figure S1).

We found three shallow clades among mtDNA sequences, suggesting divergent demographic histories that were geographically correlated (Figure S1e). Two “C” groups were found south of ~35°N, corresponding to warm temperate biogeographic provinces, whereas the “T” group was found north of ~35°N corresponding to cold temperate biogeographic provinces (Briggs & Bowen, 2011; Spalding et al., 2007). The “C” clade with 10 haplotypes was found predominately in the East China Sea and Yellow Sea, whereas the other “C” clade was found further east from South Korea to the Chiba Peninsula in Central Honshu (Figure S1). The “T” haplotypes may have spread northward from the refugium in the Sea of Japan/East Sea, whereas the “C” haplotypes were restricted to warm‐water provinces. Present‐day maintenance of the “C”/“T” boundary may reflect current patterns, such as the barrier at Cape Inubo where the warm‐water Kuroshio Current meets the cold‐water Oyashio current. It is difficult to assess which clade is most basal given the lack of sequence information for closely related *Gracilaria* species.

Although the majority of studies have focused on broader scale patterns across the marginal seas of the NW Pacific from southern China to Japan (Ni et al., 2014), there is strong genetic differentiation between populations along the Sea of Japan/East Sea and the Pacific coast. For example, the eastern and western coastlines of Japan were genetically differentiated between populations of *Pterogobus* gobies (Akihito et al., 2008), the fucoids *Sargassum hemiphyllum* (Cheang & Chung, 2010) and *S. horneri* (Uwai, Kogame, Yoshida, Kawai, & Ajisaka, 2009) and the kelp *Undaria pinnatifida* (Uwai et al., 2006), but not in the gastropod *Littorina brevicula* (Azuma & Chiba, 2015). The “T” haplotype 6 was common on both coastlines of Japan at roughly 35°N and the eastern coastlines of South Korea and Russia, but haplotypes 5 and 7 and 16, 18, and 28 were only sampled in the Sea of Japan/East Sea and Pacific Ocean populations, respectively (Figure S1a).

Microsatellite genotypes, on the other hand, were characterized by genetic differentiation that corresponded to a large degree with extant current patterns (Figures 3 and S3). For example, the southern Japanese sites, from southeastern Kyushu to the Chiba Peninsula and likely isolated by the Kuroshio Current, formed a large cluster with minimal admixture (Figure S3). Likewise, Sea of Japan/East Sea and Hokkaido sites formed a group that was consistent with the flow of the Tsushima Current. In contrast, the thalli sampled at Hayase River (hay) and Futatsuiwa (fut) were genetically more similar to northeastern Honshu sites (mou and nag). It is possible that these reflect some movement of *Gracilaria vermiculophylla* thalli as a result of aquaculture practices (i.e., oysters or the alga itself). Finally, the similarity of thalli sampled at Shikanoshima (shk) to thalli sampled from putative source sites (akk, mng, sou) is likely due to the frequent fishery transport between Hokkaido and Kyushu (M. Nakaoka, personal communication), of which similar patterns have been found in the red seaweed *Gelidium elegans* (Kim et al., 2012).

### 4.2 Pathway of the oyster?

*Gracilaria vermiculophylla* invaded low‐energy, high‐salinity estuaries dominated by soft‐sediments on all continental margins of North America (Bellorin et al., 2004; Kim et al., 2010), habitats in which oysters were seeded and cultivated in huge quantities (Ruesink et al., 2005). However, the first records of *G. vermiculophylla* along the WNA, EUSA, and EU coastlines were 1979 (Bellorin et al., 2004), 1998 (Thomsen et al., 2006), and 1995 (Rueness, 2005), respectively, which are decades after deliberate oyster movement (Barrett, 1963; Ruesink et al., 2005), leading to speculation that hull fouling or ballast water vectors were more likely (Ruiz, Fofonoff, Steves, Foss, & Shiba, 2011; J. Carlton, personal communication). Although *G. vermiculophylla* thalli have been shown to survive desiccation (Nyberg & Wallentinus, 2009), they are negatively buoyant and are unlikely to be taken up in ballast water. Moreover, red algal spermatia and spores are only viable for several hours to a few days (see Destombe, Godin, & Remy, 1990 or reviewed in Krueger‐Hadfield, Roze, Correa, Destombe, & Valero, 2015) and would not likely survive an oceanic crossing. Moreover, *G. vermiculophylla* is not commonly found on boat hulls or pontoons in marinas (see Mineur, Johnson, & Maggs, 2008; S.A. Krueger‐Hadfield, personal observation).

Our results, however, point to oyster transport as the main vector for the spread of *Gracilaria vermiculophylla*. Specifically, our genetic analyses assigned nearly all introduced algal thalli to three prominent sites of oyster aquaculture (reviewed in Barrett, 1963; Byers, 1999; Ruesink et al., 2005) along the northeastern coastline of Japan: Soukanzan (sou, Matsushima Bay), Mangoku‐ura (mng, Mangoku Bay), and Akkeshi (akk, Lake Akkeshi). Anecdotal evidence suggests that oysters introduced to Puget Sound were from Akkeshi (Galtsoff, 1932) and that subsequent introductions to California (Barrett, 1963; Chew, 1979) and France (Goulletquer, 1998; Maurin & LeDantec, 1979) were mainly from Matsushima Bay (Chew, 1979). Moreover, sequencing of herbarium specimens has pre‐dated the first records of *G. vermiculophylla* thalli by several decades along the WNA and EUSA coastlines (S.A. Krueger‐Hadfield, E.E. Sotka & K.A. Miller, unpublished data). First records now align with the same decades in which *Crassostrea gigas* was introduced to WNA and EUSA estuaries. Mineur, Le Roux, Maggs, and Verlaque (2014) used the discovery rate of non‐native species in Europe from 1966 to 2012 and suggested continuous importation of oysters over this entire period. Thus, in EU estuaries, *G. vermiculophylla* introductions are possible much earlier than currently documented. While we cannot eliminate deliberate introductions for *Gracilaria* aquaculture, or incidental introductions from hull fouling or ballast water as playing a role, the simplest, most parsimonious explanation is oyster introductions.

Surprisingly, and despite the fact that dozens of species were introduced worldwide with Japanese *Crossastrea gigas* from northeastern Japan (Byers, 1999; Ruesink et al., 2005), we know only a single study in which this region was independently identified as an invasion source using genetic markers (i.e., the mud snail *Batillaria attramentaria,* Miura et al., 2006). The dearth of studies that identified this historically important source region is due either to poor resolution of markers, poor sampling of native range populations, or both. Our study and Miura et al. (2006) rigorously sampled the native and non‐native ranges enabling the identification of intensive oyster aquaculture as the source populations, while simultaneously excluding other native sites as candidate donor regions.

While *Crassostrea gigas* has been introduced to the Southern Hemisphere (Ruesink et al., 2005), there are no records of *Gracilaria vermiculophylla* from these areas. However, there are known free‐floating populations of *Gracilaria* and *Gracilariopsis* thalli from Argentina (Martín, Boraso de Ziaxso, & Leonardi, 2011) and South Africa (Govender, 2011; J. Bolton, personal communication). Thalli in other Northern Hemisphere bays and estuaries may have been previously misidentified as native congenerics or different genera (Tomales Bay, CA: Huntington & Boyer, 2008; Bodega Bay: Grosholz & Ruiz, 2009) until representative thalli were barcoded using molecular tools (e.g., Saunders, 2009). It is possible that *G. vermiculophylla* thalli are cryptogenic in the Southern Hemisphere and simply await documentation.

### 4.3 Complexity of introduction history

There were likely multiple *Gracilaria vermiculophylla* invasions along North American, European, and northwest African shorelines. Along the WNA coast, estuaries in Tomales Bay (tmb), Elkhorn Slough (elk), Puget Sound (ptw), and British Columbia (pmo, bam) were composed of different genetic cohorts of *G. vermiculophylla*. This is consistent with the anecdotal histories of oyster introduction and cultivation in these locations (Barrett, 1963). Most WNA thalli (~83%) had a genetic signature consistent with a northeastern Japanese source, but there were thalli from California and Puget Sound/British Columbia that appeared to be a mix of northern and southern Japanese genotypes (Figure 3, Figure S3). Similarly, some Bamfield thalli (bam) were the “C” mitochondrial genotypes. Together, this suggests some movement of southern thalli did occur historically (Byers, 1999), but the northeastern coastline of Japan was the source of the overwhelming majority of non‐native thalli.

Along the EU coast, we also found evidence of genetic structure consistent with multiple introductions. There is a long and complicated history of oyster introductions that dates back to the 1600s directly from the northwest Pacific (Goulletquer, 1998; Grizel & Héral, 1991; Haydar & Wolff, 2011; Lallias et al., 2015; Ó Foighil, Gaffney, Wilbur, & Hilbish, 1998). In addition to *Crassostrea gigas* introductions, *C. virginica* was introduced from the EUSA to hatcheries and aquaculture facilities throughout Western Europe (Ruesink et al., 2005). Other invaders, such as *Sargassum muticum* and *Crepidula fornicata*, are thought to have been accidentally introduced to Europe as a result of *C. gigas* oyster introductions from Japan or British Columbia (Farnham, Fletcher, & Irvine, 1973) or *C. viriginica* imported from EUSA (Riquet, Daguin‐Thiébaut, Ballenghien, Bierne, & Viard, 2013), respectively. The similarity of European sites to some WNA and EUSA sites suggests that the invasion of the European coastline may reflect primary invasions from Japan, secondary invasions from WNA and EUSA or both. With our current set of microsatellite genotypes, we cannot at present distinguish between these alternatives.

Along the EUSA coastline, there were at least two different invasions: one to Long Island Sound (nyc, lhp) and Narragansett Bay (ris) and the other to the outer coast of Virginia and the Chesapeake Bay (Figure 3). The first introduction stayed localized to Long Island Sound to the Cape Cod Peninsula and maintains a genetic constitution that is more similar to the EU and WNA than to the other sites along the EUSA. The second introduction rapidly spread along the eastern seaboard to New Hampshire (grb) and south to the Carolinas and Georgia and may have been introduced directly from Japan with oysters (Mann, 1979). However, as with the EU, we cannot distinguish among primary and secondary invasions with our current data.

Although the first records of EUSA *Gracilaria vermiculophylla* only date back to 1998, this alga likely occurred in the Chesapeake Bay and along New England coastlines for longer than currently thought and was misidentified as the native congeneric *G. tikvahiae* (Thomsen et al., 2006; Nettleton, Mathieson, Thornber, Neefus, & Yarish, 2013; S.A. Krueger‐Hadfield & K.A. Miller, unpublished data). *Crassostrea gigas* was introduced to Maine and Massachusetts in 1949 and the 1970s (Dean, 1979; Hickey, 1979), Connecticut in the 1940s (Loosanoff & Davis, 1963), New Jersey in the 1930s, Delaware in 1962, and Maryland in the 1970s (Andrews, 1980). Likewise, the arrival of an oyster pathogen in the Chesapeake Bay was linked to rogue plantings of *C. gigas* from Japan, California, the northwest Pacific, or some combination of these oyster cultivation regions (Burreson, Stokes, & Friedman, 2000). Taken together, our contemporary genetic data strongly point to the movement of oysters as the main vector and pathway for moving *Gracilaria vermiculophylla* across oceanic basins.

### 4.4 Secondary spread within coastlines

Our genetic data suggest different patterns of secondary spread once *Gracilaria vermiculophylla* invaded the three non‐native coastlines of the Northern Hemisphere. Along the WNA coastline, the genetic structure of *G. vermiculophylla* was the most profound, relative to other continental margins. This suggests that multiple primary invasions arrived from Japan and that secondary spread was likely less important than along the EUSA and EU coastlines. This is a tentative conclusion, however, as the geographic extent of the introduction of *G. vermiculophylla* is poorly characterized along the WNA coast. In 2015, for example, we found *G. vermiculophylla* in Bodega Bay (bob), Tomales Bay (tmb), and San Diego (E.E. Sotka and S.A. Krueger‐Hadfield, unpublished data). Grosholz and Ruiz (2009) documented the dramatic change in abundance of what they called *Gracilariopsis sjoestedtii* in Bodega Harbor as a result of the invasion of the European crab *Carcinus maenas*. They analyzed aerial photographs, but performed their study in the same area we sampled *G. vermiculophylla* in this study. An herbarium sample at the Bodega Marine Lab from the late 1960s from this same area was labeled *Gracilaria* cf. *vermiculophylla* (P.G. Connors, personal communication) and has subsequently been identified as *G. vermiculophylla* (S.A. Krueger‐Hadfield, E.E. Sotka & K.A. Miller, unpublished data).

Relative to the case along the WNA coastline, the magnitude of secondary spread along the EU and EUSA coastlines is less speculative. Along the EUSA, the high levels of diversity observed in the sites studied along the eastern shore of Virginia (Figure 4; see also Gulbransen et al., 2012) strongly suggest that the outer coast of Virginia and the Chesapeake Bay were the initial sites of introduction for the larger EUSA invasion. Rapid colonization southward has been documented, such as throughout the estuaries of South Carolina (Byers et al., 2012), possibly facilitated by the Intracoastal Waterway and movement of fishing vessels or leisure craft (Freshwater et al., 2006). Consistent with this southward invasion pathway, there was a decline in allelic diversity from Virginia southward to Georgia (Figure 4).

Along the coastlines of the British Isles, Europe, and northern Africa, our data are consistent with multiple invasion events into France and Portugal, followed by secondary spread to other estuaries. In the Marennes‐Oléron basin, for example, *G. vermiculophylla* thalli from Loix (frl) and Marennes (fme) belonged to different genetic cohorts (Figure S3). This basin is an area of intense aquaculture. The similarity of the sites sampled in Brittany (fdm, pol, fpa) to the thalli sampled at Marennes (fme) may be the result of secondary spread as oysters are commonly moved throughout French cultivation facilities in the English Channel, Bay of Biscay, and Mediterranean throughout their life cycle (Goulletquer, 1998). By contrast, sites sampled along the Jutland Peninsula (hei, dhd, nib, nor, man) were largely composed of a single genetic cluster from *K *=* *2 to *K *=* *23 (Figure 3, Figure S3) and exhibited lower estimates of diversity than other European sites (Figure 4). Gracilarioid seaweeds were not found in the Kiel fjord, for example, until the mid‐2000s, suggesting the invasion of Jutland estuaries is more recent (F. Weinberger, personal observation). These sites also exhibited genetic signatures of extensive vegetative fragmentation where we previously found few unique genotypes (Krueger‐Hadfield et al., 2016). On the other hand, oysters have repeatedly been introduced into the German Wadden Sea from Portugal (Mayer‐Waarden, 1964), which was reflected in the genetic similarity of the Jutland populations with those of the Ria d'Aveiro in Portugal (Gafanha/gaf and Estarreja/est; Figure S5a). We did not sample Mediterranean populations, which may help to identify pathways, both primary and secondary, that are cryptic in this current study, such as the introduction of the Manila clam (*Venerupis philippinarum*) to the Mediterranean (Sfriso, Maistro, Andreoli, & Moro, 2010).

The vector of secondary spread along the EU and EUSA coastlines is uncertain. Natural current patterns may contribute to the natural spread of thalli. Spread of *Crassostrea gigas* throughout the East Frisian Wadden Sea may be responsible for the encroachment of *Gracilaria vermiculophylla* along the Jutland Peninsula (Schmidt et al., 2008). Indeed, *G. vermiculophylla* is often found near oyster farms (Rueness, 2005). Other studies have documented the arrival of non‐native species as a result of leisure craft (e.g., Griffith et al., 2009); however, we have not found *G. vermciulophylla* on boat hulls or pontoons (S.A. Krueger‐Hadfield*,* personal observation). Nevertheless, some EU sites, such as Heiligenhafen (hei), were near marinas and could point to accidental within‐coastline transport by sailboats. Nyberg and Wallentinus (2009) suggested migrating seabirds may also be responsible for the transport of small fragments of *G. vermiculophylla*. Seabird sanctuaries in closed lagoons along the German Baltic Sea coast that are inaccessible to aquaculture, boat traffic, and other human activities often harbor isolated populations of *G. vermiculophylla* (F. Weinberger and M. Hammann, personal observation). Birds have also been known to use gracilarioid seaweeds in nests (Arnold, 1968), including *G. vermiculophylla* (S.A. Krueger‐Hadfield and S.J. Shainker, personal observation). Along the EUSA, spread of *G. vermiculophylla* may be via shrimp nets and crab pots, as *G. vermiculophylla* is known to be a nuisance in some estuaries to shrimpers (Freshwater et al., 2006).

## 5 Conclusions

*Gracilaria vermiculophylla* is now recognized as one of the most widespread and abundant marine invaders in the Northern Hemisphere (Kim et al., 2010; Krueger‐Hadfield et al., 2016; Saunders, 2009) and has transformed the ecosystems to which it has been introduced (Byers et al., 2012; Thomsen, Wernberg, Tuya, & Silliman, 2009). Nevertheless, this cryptic invader has likely been lurking in estuaries throughout the Northern Hemisphere for decades without recognition (S.A. Krueger‐Hadfield, E.E. Sotka and K.A. Miller, unpublished data). This oversight may reflect the fact that phycologists are more likely to study rocky shores than soft‐sediment estuaries where *G. vermiculophylla* has largely invaded, or the fact that cryptic species are common in seaweeds (Krueger‐Hadfield, Magill, Mieszkowska, Sotka, & Maggs, 2017). An analogous example comes from Provan, Booth, Todd, Beatty, and Maggs (2007), who used herbarium specimens coupled with molecular markers to conclude that the invasive strain of *Codium fragile* spp. *tomentosoides* colonized sites around the world for at least 100 years longer than previously reported from contemporary sampling.

Our identification of a source region has implications for understanding the evolutionary ecology of the *Gracilaria vermiculophylla* invasion, in particular. Previous studies have suggested introduced populations evolved greater tolerance for heat stress and resistance to herbivory during the invasion (Hammann, Wang, Boo, Aguilar‐Rosas, & Weinberger, 2016; Hammann, Wang, Rickert, Boo, & Weinberger, 2013). However, these studies compared introduced populations with South Korean and Chinese native populations that do not represent the source of the invasion. If there is geographic variation in the native range in these traits, then it is possible that the source populations had already evolved these traits before the invasion (Bossdorf, Lipowsky, & Prati, 2008; Colautti & Lau, 2015; Estoup & Guillemaud, 2010).

More broadly, Carlton (2009) argued convincingly that fewer assumptions should be made about the status of a given taxon (e.g., native or cryptogenic) without appropriate morphological and molecular analyses. This is particularly true for understudied groups in under‐explored habitats, such as macroalgae in estuarine habitats. We extend Carlton's (2009) suggestion to thoroughly explore not only the systematics of invaders, but also invasion vectors and pathways. Fewer assumptions should be made about the likelihood of a given vector without appropriate historical and contemporary genetic sampling and analyses.

## Conflict of Interest

None declared.

## Author Contributions

SAKH, NMK, and EES conceived the study; SAKH, NMK, JEB, MH, SJS, RT, FW, and EES collected samples; SAKH, NMK, and SJS extracted DNA; SAKH, NMK, SJS, TWG, and DM generated data; SAKH, AES, and EES analyzed data; JEB and AES contributed to discussions; SAKH and EES wrote the manuscript; all authors approved the final manuscript.
