# Supplementary material for: Genetic identification of source and likely vector of a widespread marine invader
Source: Ecol Evol. 2017 May 11;7(12):4432–47. doi: 10.1002/ece3.3001 (PMC5478068; doi:10.1002/ece3.3001)

GENETIC IDENTIFICATION OF SOURCE AND LIKELY VECTOR OF A WIDESPREAD MARINE INVADER

Stacy A. Krueger-Hadfield^1,2*^, Nicole M. Kollars^2†^, Allan E. Strand^2^, James E. Byers^3^, Sarah J. Shainker^2^, Ryuta Terada^4^, Thomas W. Greig^5^, Mareike Hammann^6^, David C. Murray^2^, Florian Weinberger^6^, Erik E. Sotka^2*^

^1^ Department of Biology, University of Alabama at Birmingham, Birmingham, AL 35294-1170, USA

^2^ Grice Marine Laboratory and the Department of Biology, College of Charleston, 205 Fort Johnson Rd, Charleston, SC 29412.

^3^ Odum School of Ecology, University of Georgia, 130 E. Green St., Athens, GA 30602.

^4^ United Graduate School of Agricultural Sciences, Kagoshima University, Korimoto 1-21-24, Kagoshima City, 890-0065, Japan ^5^ NOAA/National Ocean Service, Center for Coastal Environmental Health and Biomolecular Research, 219 Fort Johnson Rd, Charleston, SC 29312.

^6^ GEOMAR Helmholtz-Zentrum für Ozeanforschung Kiel, Düsternbrooker Weg 20, D-23105 Kiel, Germany.

*Corresponding authors: Stacy A. Krueger-Hadfield [sakh@uab.edu](mailto:sakh@uab.edu); Erik E. Sotka eriksotka@gmail.com

†Current address: Center for Population Biology, University of California, Davis, CA, 95616.

**Figure S1.** Mitochondrial *cox*1 haplotypic diversity from Kim et al.*,* (2010) and this study. For maps and the tree, red and blue haplotypes have either a T or a C, respectively, at the 945^th^ bp. Sites sampled across the known distribution of *Gracilaria vermiculophylla*: a) Native range, b) Non-native range west coast of North America, c) Non-native range east coast of the United States and d) Non-native range Europe and northern Africa. Haplotypes are shown next to the site in which they were sampled by Kim et al., (2010; shown in bold) and this study (shown in bold, italics and underlined). e) A phylogenetic tree of haplotypes from Kim et al., (2010) and this study. f) A 1.5% agarose gel with restriction enzyme products for six native non-source individuals from Odo-2-ri in Korea and Qingdao in China (C’s) and four individuals from Fort Johnson in the Charleston Harbor (T’s). These individuals were used as controls for all assays.

a)

b)


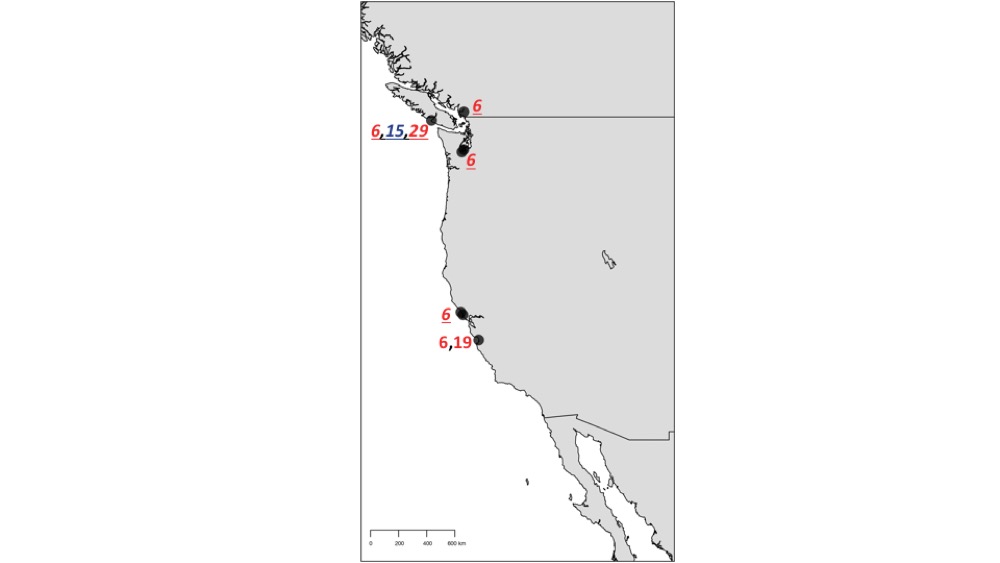


c)


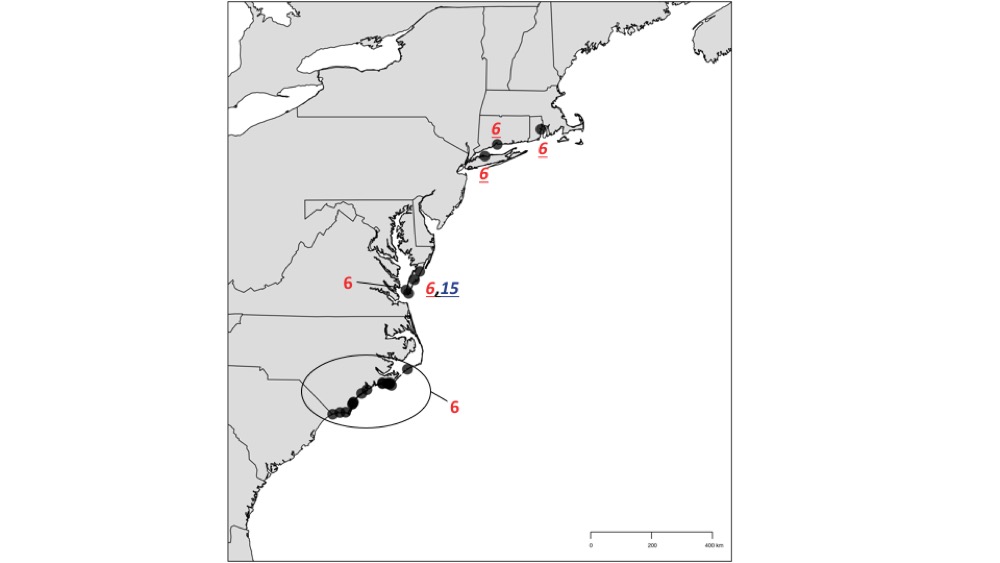


d)


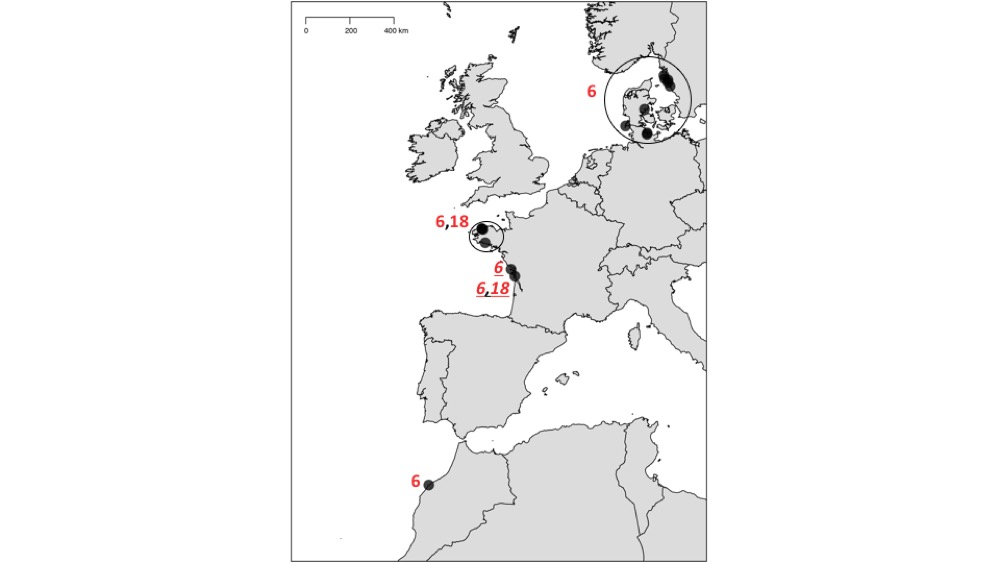


e)

f)


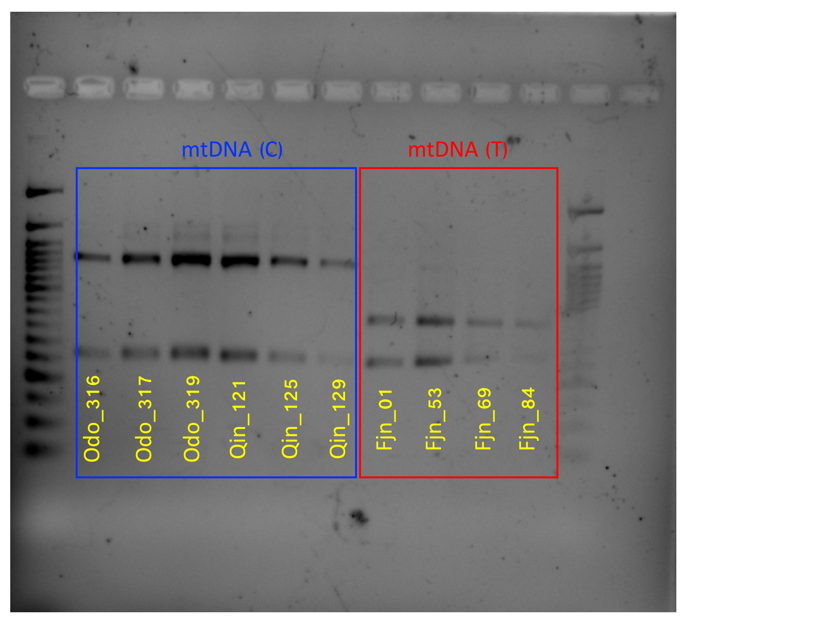


**Table S2.** a) The polymorphic sites across the 19 haplotypes from Kim et al., (2010) and the new haplotypes described in this study across the native and non-native ranges of *Graclaria vermiculophylla*. The SNP that differentiates the native source and non-source regions is shaded in gray. We used the haplotype numbers described by Kim et al., (2010), but not the ones described in Gulbransen et al., (2012). In the latter study, new haplotype numbers in Virginia were assigned based on aligning sequences across different studies and from sequences that did not span the entire length of the sequence (i.e., from 43F to 1549R). However, to avoid confusion with previously published haplotype numbers by Gulbransen et al., (2012), we have chosen different numbers for the six new haplotypes described here. Two of the Kim et al., (2010) haplotypes shared the same GenBank acc. Number (GU907106, see JPY_905_sm_TableS1-2). We called acc. GU907107 Haplotype 7 from Donghae (G530) and acc. GU907106 Haplotype 12 from Jeju (G510). b) The sites in this study for which *cox*1 was sequenced and the number of thalli at each site belonging to each haplotype.

a)

|  | **51** | **98** | **110** | **156** | **158** | **287** | **296** | **317** | **329** | **347** | **377** | **380** | **382** | **398** | **413** | **503** | **611** |
| --- | --- | --- | --- | --- | --- | --- | --- | --- | --- | --- | --- | --- | --- | --- | --- | --- | --- |
| Haplotype 01 (GU907110) | T | A | C | G | C | T | T | C | C | T | C | G | C | A | A | A | C |
| Haplotype 02 (EF434936) | . | . | . | . | T | . | . | . | . | . | . | . | . | . | . | . | . |
| Haplotype 03 (GU907108) | . | . | . | . | T | . | . | . | . | . | . | . | . | . | . | G | . |
| Haplotype 04 (GU907109) | . | . | . | . | T | C | . | . | . | . | . | . | . | G | . | . | . |
| Haplotype 05 (EF434926) | . | . | . | . | T | A | . | . | . | . | . | . | . | G | . | . | . |
| Haplotype 06 (EF434927) | . | . | . | . | T | . | . | . | . | . | . | . | . | G | . | . | . |
| Haplotype 07 (GU907107)* | . | . | . | . | T | . | . | . | . | . | . | . | T | G | . | . | . |
| Haplotype 08 (EF434929) | . | . | . | A | T | . | . | T | . | . | . | . | . | G | . | . | . |
| Haplotype 09 (GU907111) | . | . | . | . | T | . | . | . | . | . | . | T | . | . | . | . | . |
| Haplotype 10 (GU907112) | . | . | . | . | T | . | C | . | . | . | . | . | . | . | . | . | . |
| Haplotype 11 (EF434935) | . | . | . | . | T | . | . | . | . | . | . | . | . | . | . | . | . |
| Haplotype 12 (GU907106)* | . | . | T | . | T | . | . | . | . | . | . | . | . | . | . | . | T |
| Haplotype 13 (GU907105) | . | . | . | . | T | . | . | T | . | . | . | . | . | G | . | . | . |
| Haplotype 14 (EF434938) | . | . | . | . | T | . | . | . | . | . | . | . | . | G | G | . | . |
| Haplotype 15 (GU907104) | . | . | . | . | T | . | . | . | . | . | . | . | . | G | G | . | . |
| Haplotype 16 (EF434937) | . | . | . | . | T | . | . | . | . | . | . | . | . | G | G | . | . |
| Haplotype 17 (GU907103) | . | . | . | . | T | . | . | . | . | C | . | . | . | . | . | . | . |
| Haplotype 18 (GU907102) | . | . | T | . | T | . | . | . | . | . | . | . | . | G | . | . | . |
| Haplotype 19 (GU907113) | . | . | . | . | T | . | . | . | . | . | . | . | . | G | . | . | . |
| Haplotype 26 (KY621338) | . | . | . | . | T | . | . | . | T | . | A | . | . | G | . | . | . |
| Haplotype 27 (KY621339) | . | G | . | . | T | . | . | . | . | . | . | . | . | G | G | . | . |
| Haplotype 28 (KY621340) | . | . | . | . | T | . | . | . | . | . | . | . | . | G | . | . | . |
| Haplotype 29 (KY621341) | . | . | . | . | T | . | . | . | . | . | . | . | . | G | . | . | . |
| Haplotype 30 (KY621342) | C | . | . | . | T | . | . | . | . | . | . | . | . | . | . | . | . |
| Haplotype 31 (KY621343) | . | . | . | . | T | . | . | . | . | . | . | . | . | . | . | . | . |

|  | **629** | **638** | **677** | **767** | **770** | **860** | **866** | **917** | **945** | **947** | **989** | **1007** | **1031** | **1040** | **1106** | **1119** | **1154** |
| --- | --- | --- | --- | --- | --- | --- | --- | --- | --- | --- | --- | --- | --- | --- | --- | --- | --- |
| Haplotype 01 (GU907110) | T | T | C | C | C | T | A | T | C | A | C | T | G | C | G | A | A |
| Haplotype 02 (EF434936) | . | . | . | . | . | . | . | . | . | . | . | . | . | . | . | . | . |
| Haplotype 03 (GU907108) | . | . | . | . | . | . | . | . | . | . | . | . | . | . | . | . | . |
| Haplotype 04 (GU907109) | . | . | . | . | . | C | . | . | . | G | T | . | . | . | T | . | G |
| Haplotype 05 (EF434926) | . | . | . | . | . | . | . | . | T | . | . | . | . | T | . | . | . |
| Haplotype 06 (EF434926) | . | . | . | . | . | . | . | . | T | . | . | . | . | T | . | . | . |
| Haplotype 07 (GU907107)* | . | . | . | . | . | . | . | . | T | . | . | . | . | T | . | . | . |
| Haplotype 08 (EF434929) | . | . | . | . | . | C | . | . | . | . | T | . | . | . | T | C | G |
| Haplotype 09 (GU907111) | . | . | . | . | . | . | . | . | . | . | . | . | . | . | . | . | . |
| Haplotype 10 (GU907112) | . | . | . | . | . | . | . | . | . | . | . | . | . | . | . | . | . |
| Haplotype 11 (EF434935) | . | . | . | . | . | . | . | . | . | . | . | . | A | . | . | . | . |
| Haplotype 12 (GU907106)* | . | . | . | . | . | . | . | . | . | . | . | . | . | . | . | . | . |
| Haplotype 13 (GU907105) | . | . | . | . | . | C | . | . | . | . | T | . | . | . | T | . | G |
| Haplotype 14 (EF434938) | . | . | T | . | . | C | . | . | . | . | T | . | . | . | T | . | G |
| Haplotype 15 (GU907104) | . | . | . | . | . | C | . | . | . | . | T | . | . | . | T | . | G |
| Haplotype 16 (EF434937) | . | . | . | . | . | . | . | . | T | . | T | . | . | T | . | . | G |
| Haplotype 17 (GU907103) | . | . | . | . | . | . | . | . | . | . | . | . | . | . | . | . | . |
| Haplotype 18 (GU907102) | . | . | . | . | . | . | . | . | T | . | . | . | . | T | . | . | . |
| Haplotype 19 (GU907113) | . | . | . | . | . | . | G | . | T | . | . | . | . | T | . | . | . |
| Haplotype 26 (KY621338) | . | C | . | T | . | . | . | . | . | . | . | . | . | . | . | . | . |
| Haplotype 27 (KY621339) | C | . | . | . | . | C | . | . | . | . | T | . | . | . | T | . | G |
| Haplotype 28 (KY621340) | . | . | . | . | T | . | . | . | T | . | . | . | . | T | . | . | . |
| Haplotype 29 (KY621341) | . | . | . | . | . | . | . | . | T | . | . | C | . | T | . | . | . |
| Haplotype 30 (KY621342) | . | . | . | . | . | . | . | . | . | . | . | . | . | . | . | . | . |
| Haplotype 31 (KY621343) | . | . | . | . | . | . | . | C | . | . | . | . | . | . | . | . | . |

b)

|  | **Haplotypes** | | | | | | | | | |
| --- | --- | --- | --- | --- | --- | --- | --- | --- | --- | --- |
| **Site** | **2** | **6** | **15** | **18** | **26** | **27** | **28** | **29** | **30** | **31** |
| akk | . | 10 | . | . | 3 | . | . | . | . | . |
| fut | . | 7 | . | . | . | . | . | . | . | . |
| hik | . | . | . | . | . | 7 | . | . | . | . |
| hit | . | 6 | . | . | . | . | 1 | . | . | . |
| mng | . | 7 | . | 1 | . | . | . | . | . | . |
| mou | . | 11 | . | . | . | . | . | . | . | . |
| nag | . | 8 | . | . | . | . | . | . | . | . |
| shk | 8 | . | . | . | . | . | . | . | . | . |
| shr | . | 8 | . | . | . | . | . | . | . | . |
| sar | . | 6 | . | . | . | . | . | . | . | . |
| usu | . | 9 | . | . | . | . | . | . | . | . |
| waj | 2 | . | . | . | . | . | . | . | 5 | 1 |
| bam | . | 4 | 3 | . | . | . | . | 5 | . | . |
| bob | . | 7 | . | . | . | . | . | . | . | . |
| eld | . | 7 | . | . | . | . | . | . | . | . |
| moo | . | 7 | . | . | . | . | . | . | . | . |
| ptw | . | 7 | . | . | . | . | . | . | . | . |
| tmb | . | 7 | . | . | . | . | . | . | . | . |
| gar | . | 4 | 4 | . | . | . | . | . | . | . |
| lhp | . | 8 | . | . | . | . | . | . | . | . |
| mac | . | 5 | 1 | . | . | . | . | . | . | . |
| mag | . | 5 | 1 | . | . | . | . | . | . | . |
| nyc | . | 7 | . | . | . | . | . | . | . | . |
| ris | . | 8 | . | . | . | . | . | . | . | . |
| fme | . | 2 | . | 6 | . | . | . | . | . | . |
| frl | . | 4 | . | . | . | . | . | . | . | . |

**Figure S2.** Discriminant analyses of principal components (DAPC). a) Cross-validation using the *xvalDapc* function as implemented in *adegenet*. The optimal number of principal components to retain was 88. b) Using the *compoplot* function as implemented in *adegenet*, we determined the *a priori* assignment of five subregions (native mtDNA-C, native mtDNA-T, WNA, EUSA and EU). There was high a probability of assignment to these *a priori* clusters (92%). The y-axis shows the membership probability and along the x-axis are the 1670 diploid thalli genotyped. The majority of thalli were assigned to their cluster: native mtDNA-C (blue) had 94% reassignment; native mtDNA-T (red) had 85.3% reassignment; WNA (purple) had 74.6.4% reassignment; EUSA (orange) had 97% reassignment; EU (pink) had 92.3% reassignment.

a)


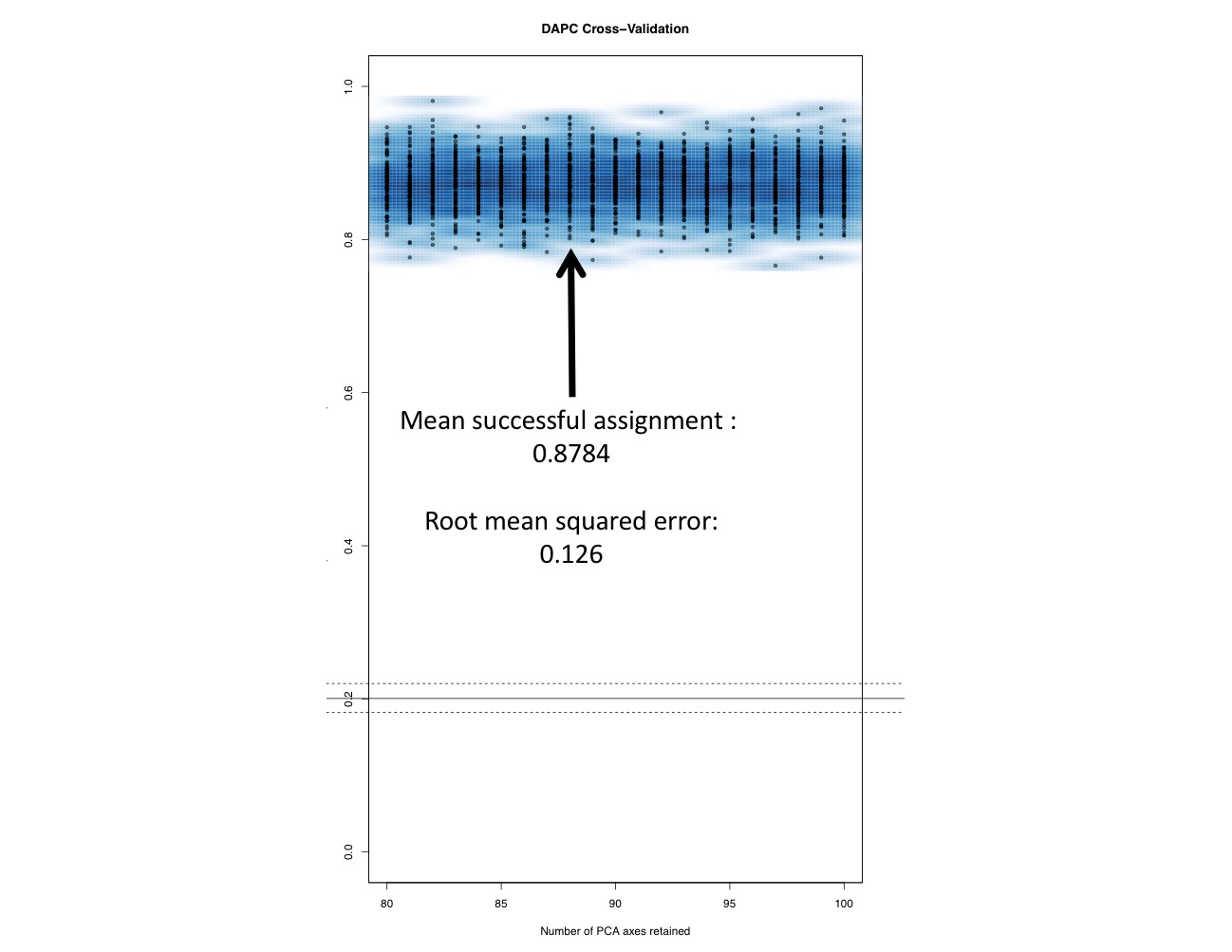


b)


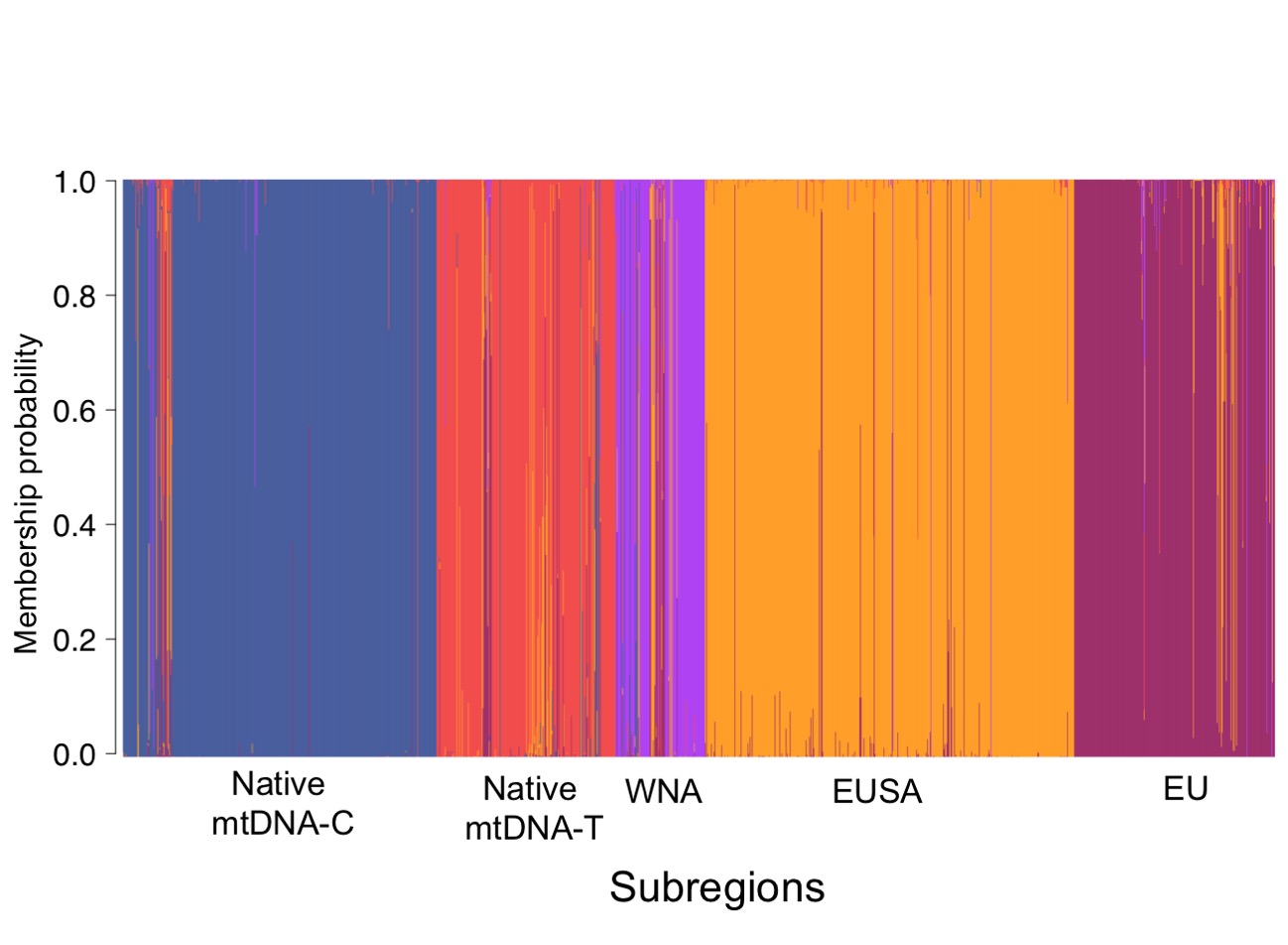


**Figure S3.** a) The number of clusters inferred for the data set of diploid thalli including one copy of each genotype based on *P_sex_*. The DIC scores + SE for *K*=2 to *K*=30 using *instruct*. The mean similarity scores among different runs at the same *K* were computed using *clumpak*. The optimal number of clusters as determined by DIC was *K* = 23, but the mean similarity score was only 0.901. b-f) Cluster assignment as inferred by *instruct* and visualized using *clumpak* for optimal alignment of the 20 independent runs for different *K*’s. The x-axis is arranged by site across the native range (divided into ‘C’ Haplotypes and ‘T’ Haplotypes) and the non-native range (divided into WNA, EUSA and EU). Each of the sites grouped together based on genetic similarity are shown by a dashed line along the x-axis (see Figure 3). b) *K* = 2. The mean similarity score was 0.995 and all 20 runs made up the major modes detected by *clumpak*. The y-axis shows the proportion of each site that belongs to a given genetic cluster. c) *K* = 3. The mean similarity score was 0.992 and 17 of the 20 runs made up the major modes detected by *clumpak*. d) *K* = 4. The mean similarity score was 0.990 and 18 of the 20 runs made up the major modes detected by *clumpak*. e) *K* = 5. The mean similarity score was 0.986 and all 20 runs made up the major modes detected by *clumpak*. f) *K* = 23. The mean similarity score was 0.901 and 19 of the 20 runs made up the major modes detected by *clumpak.* The site groupings based on genetic similarity shifted with the increased number of genetic clusters so that some sites that were previously grouped were no longer dominated by the same genetic cluster (e.g., dae and gye), whereas, other sites became genetically more similar (e.g., don, usu and jon).

a)

b)


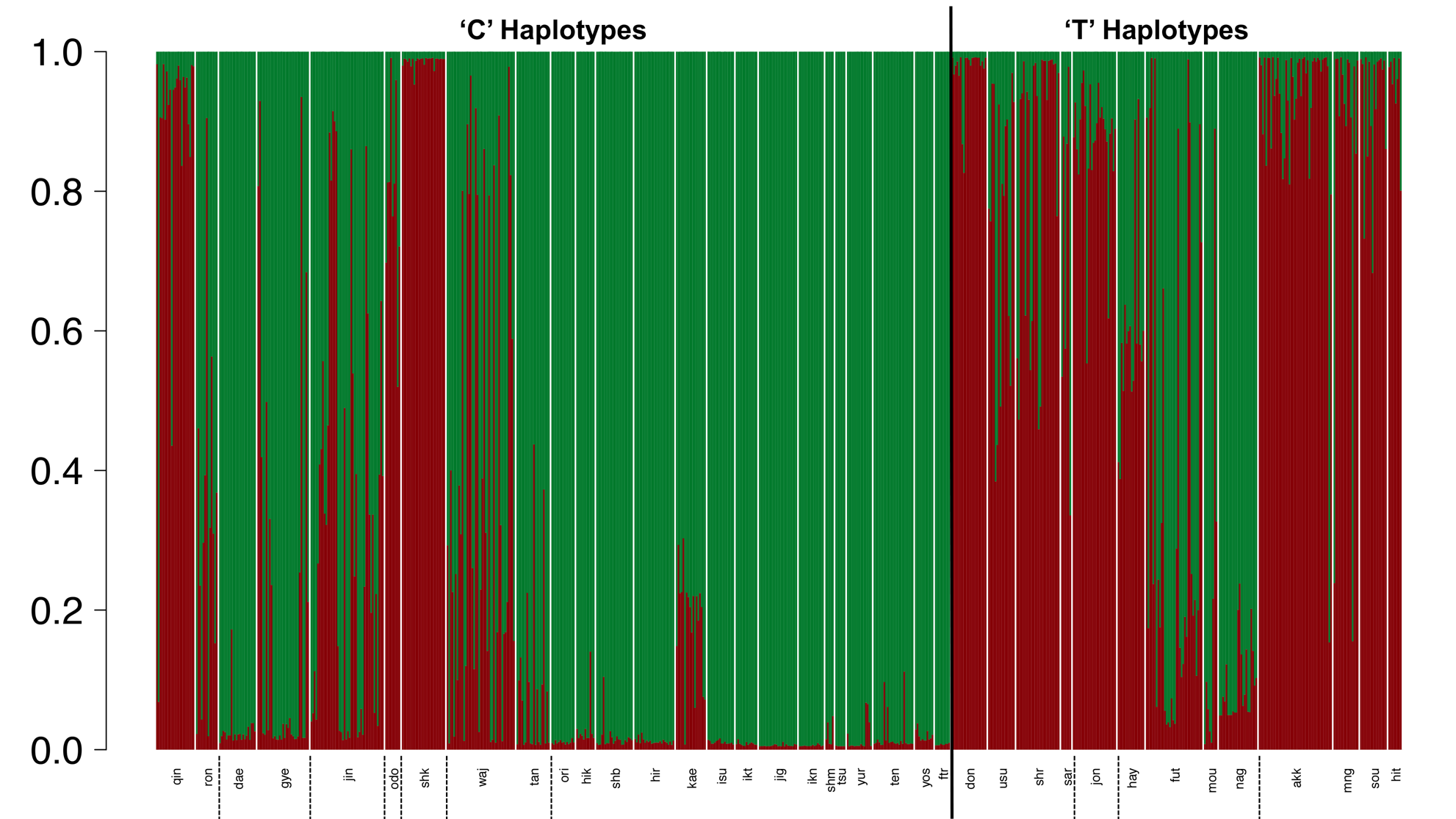


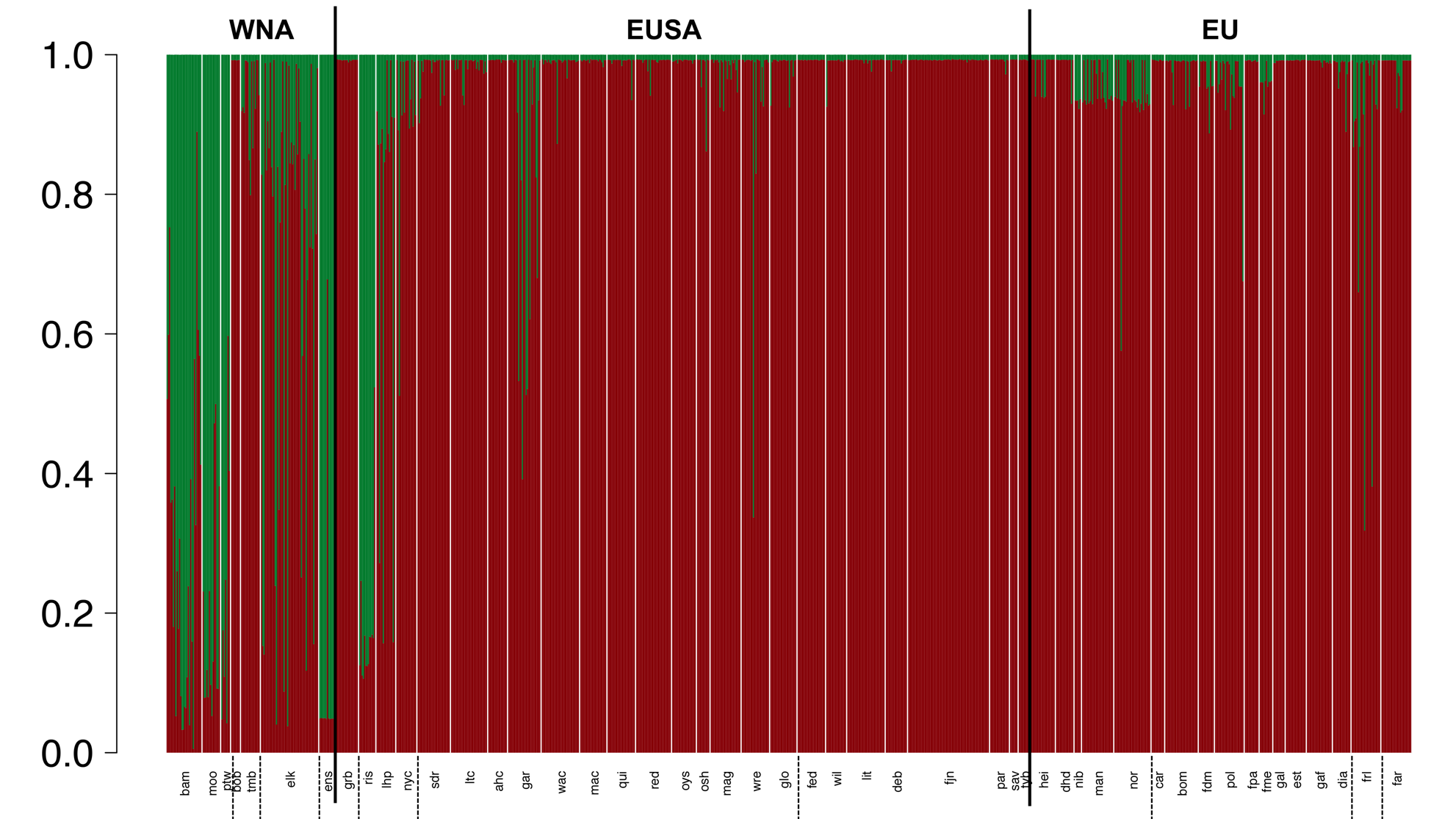


c)


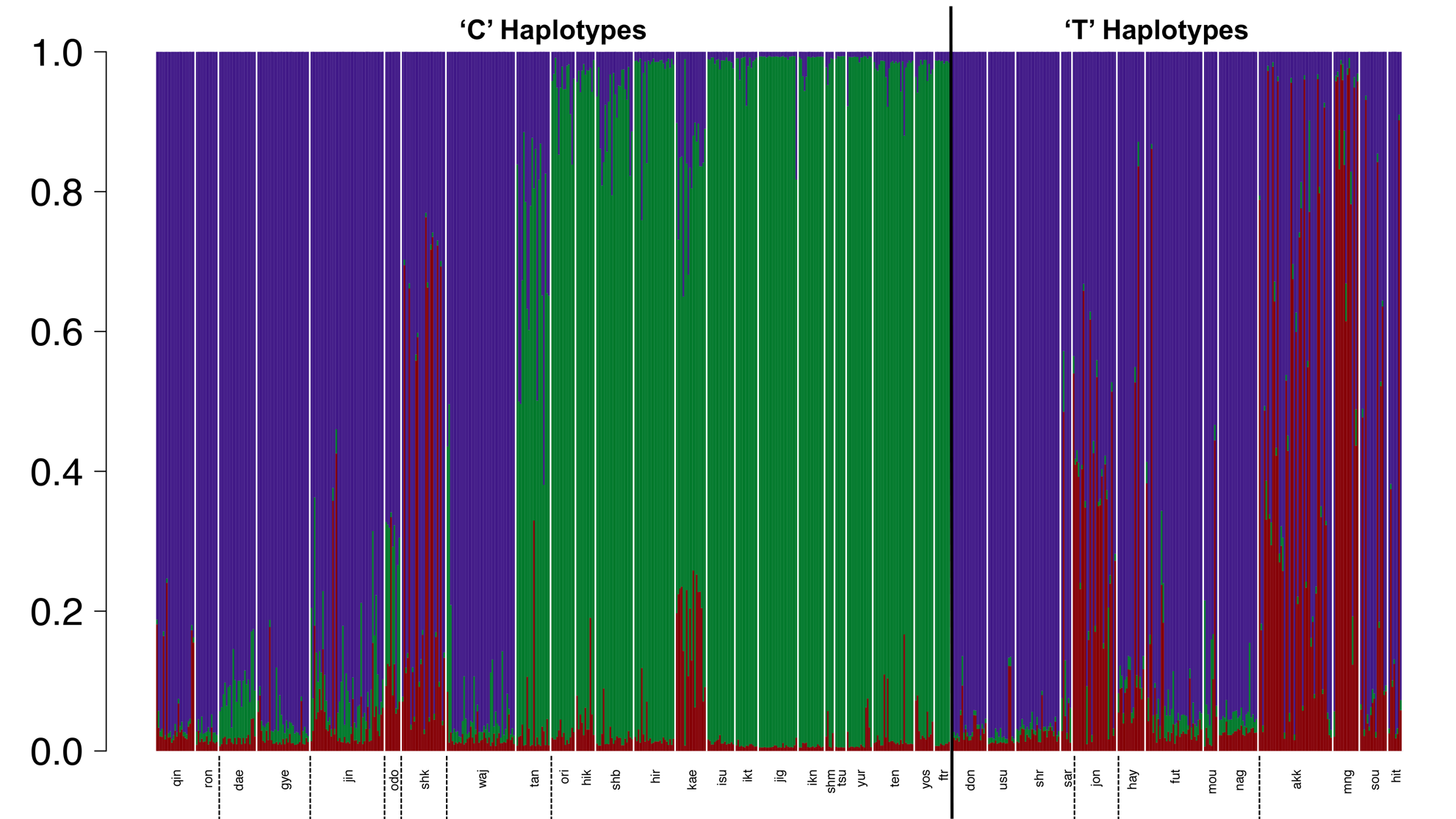


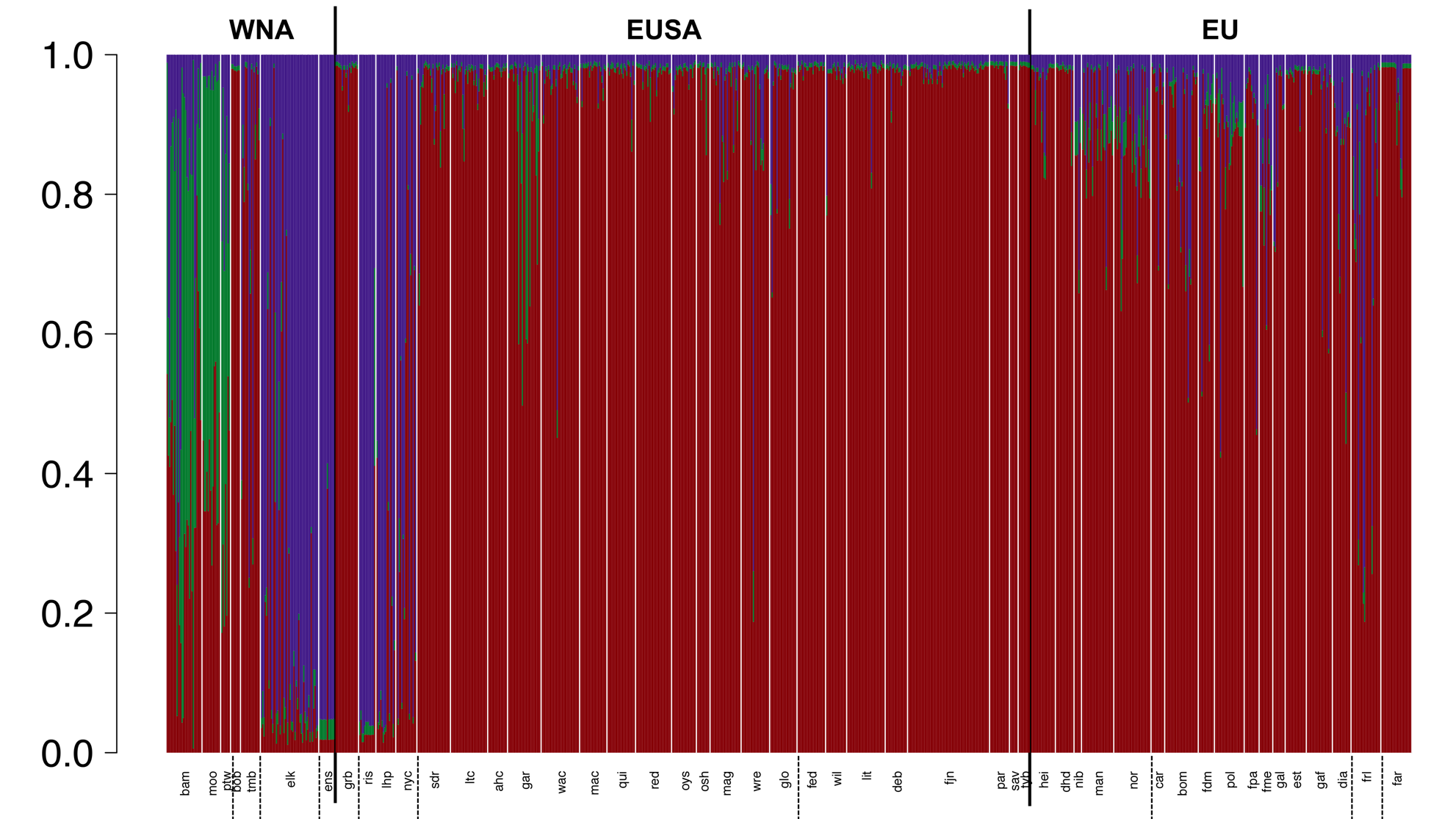


d)


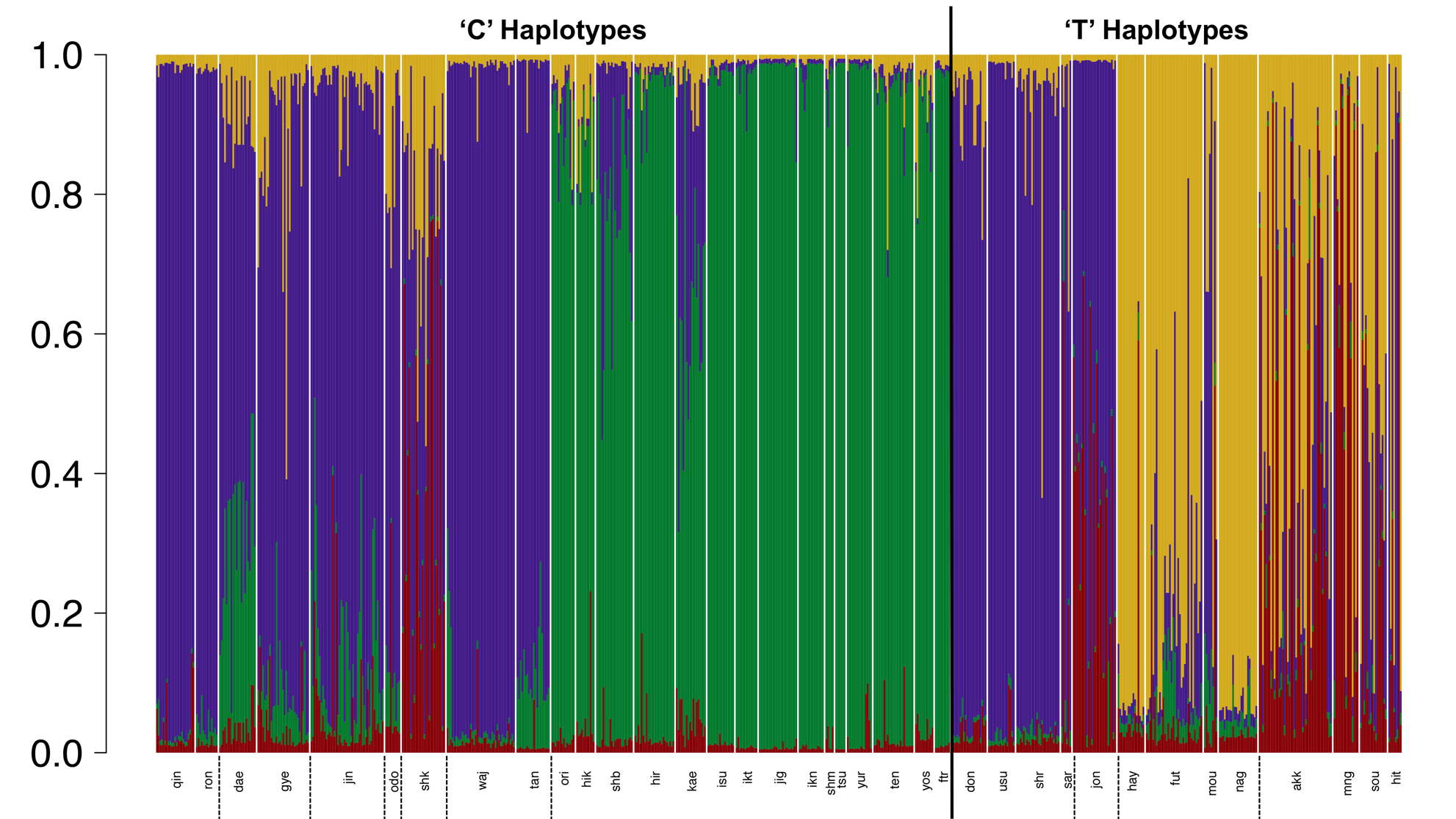


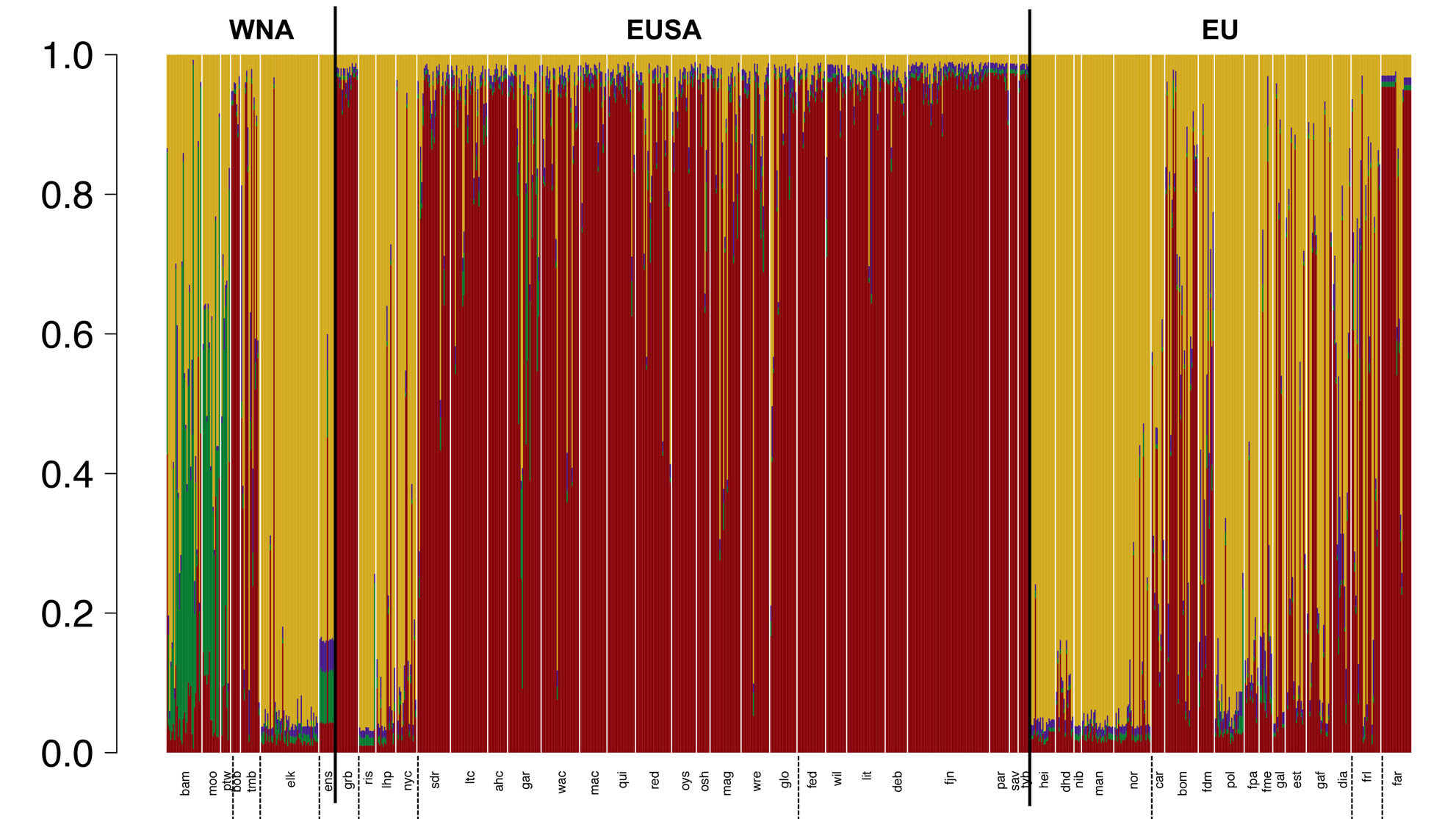


e)


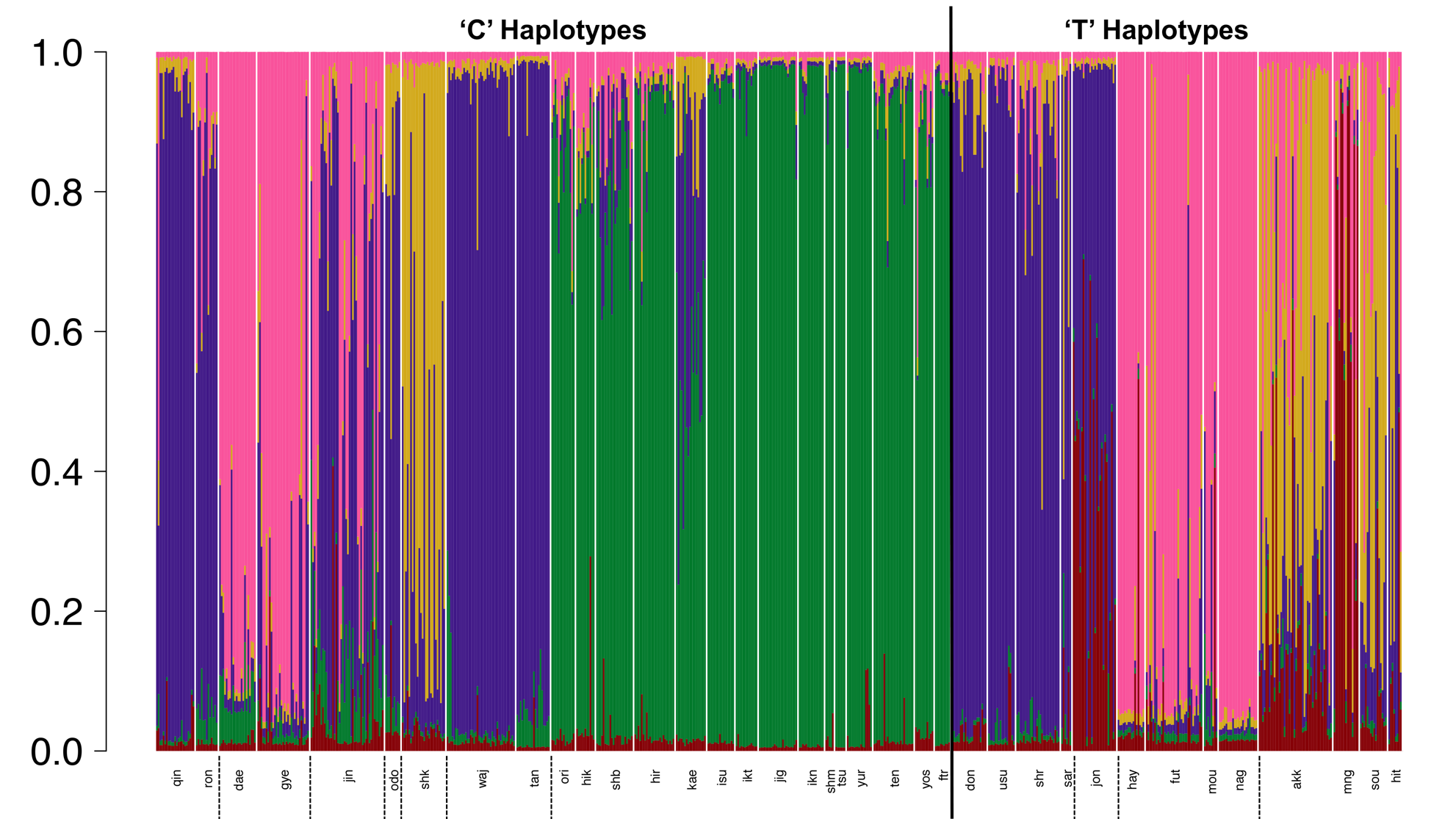


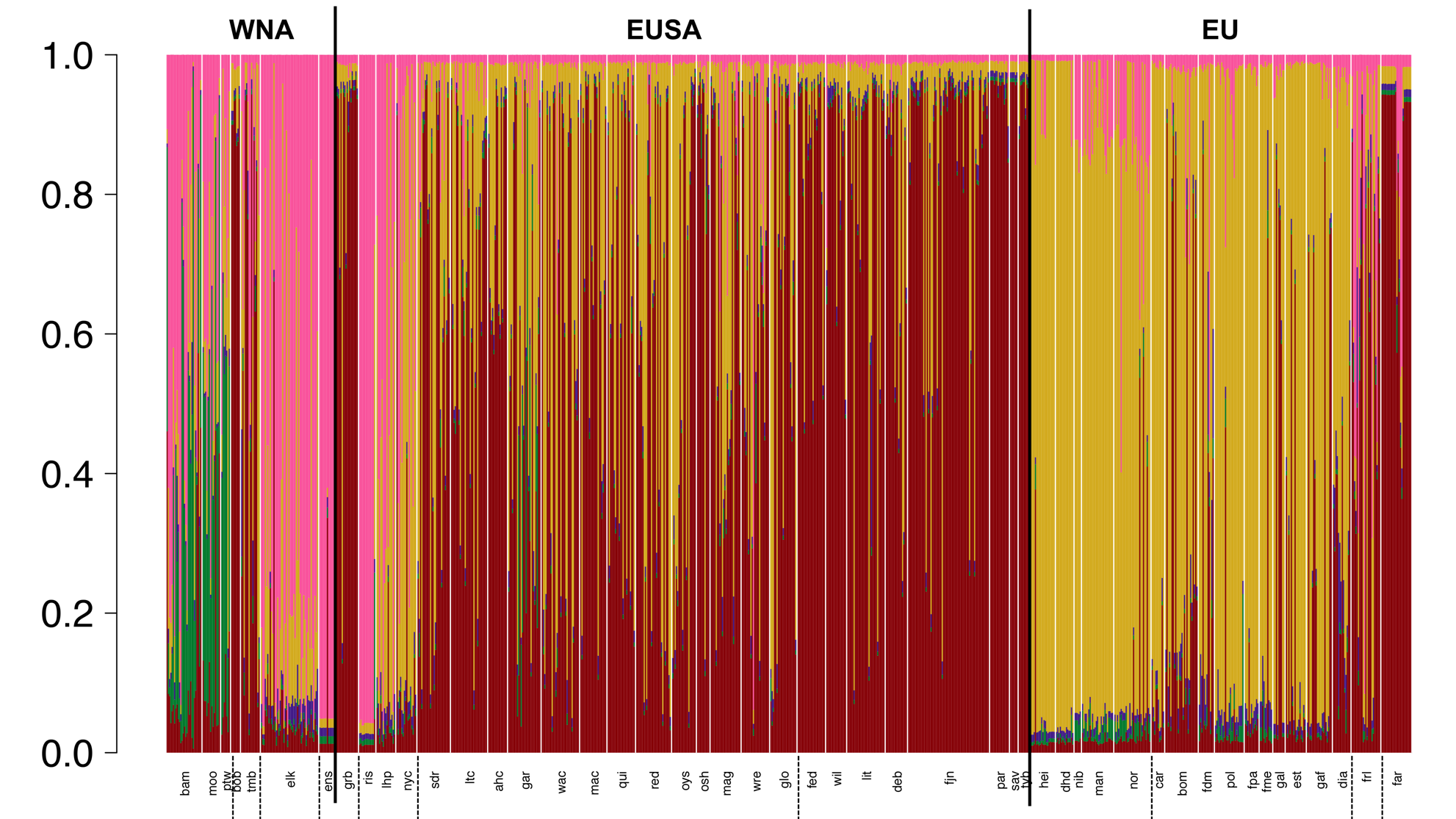


f)


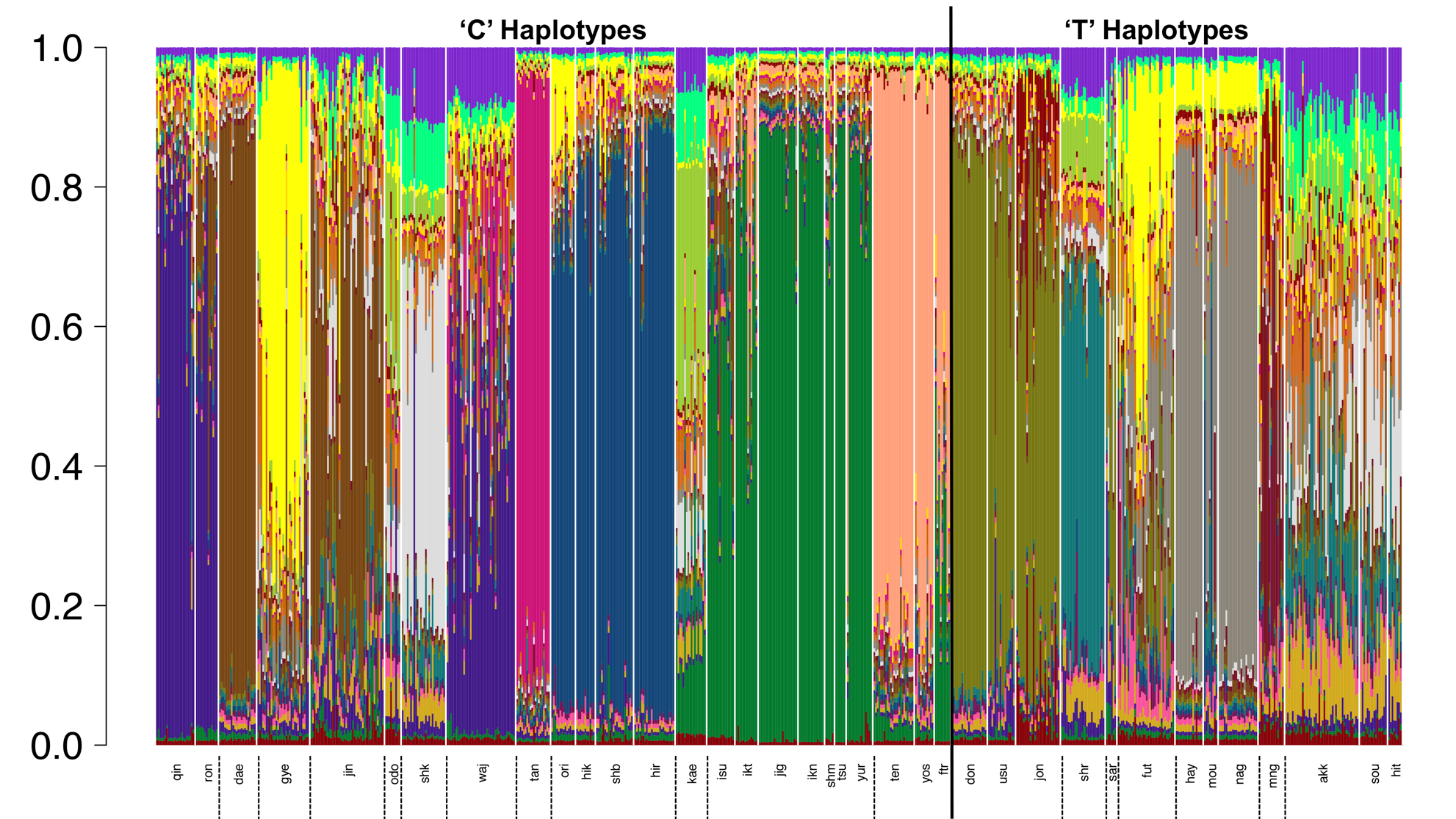


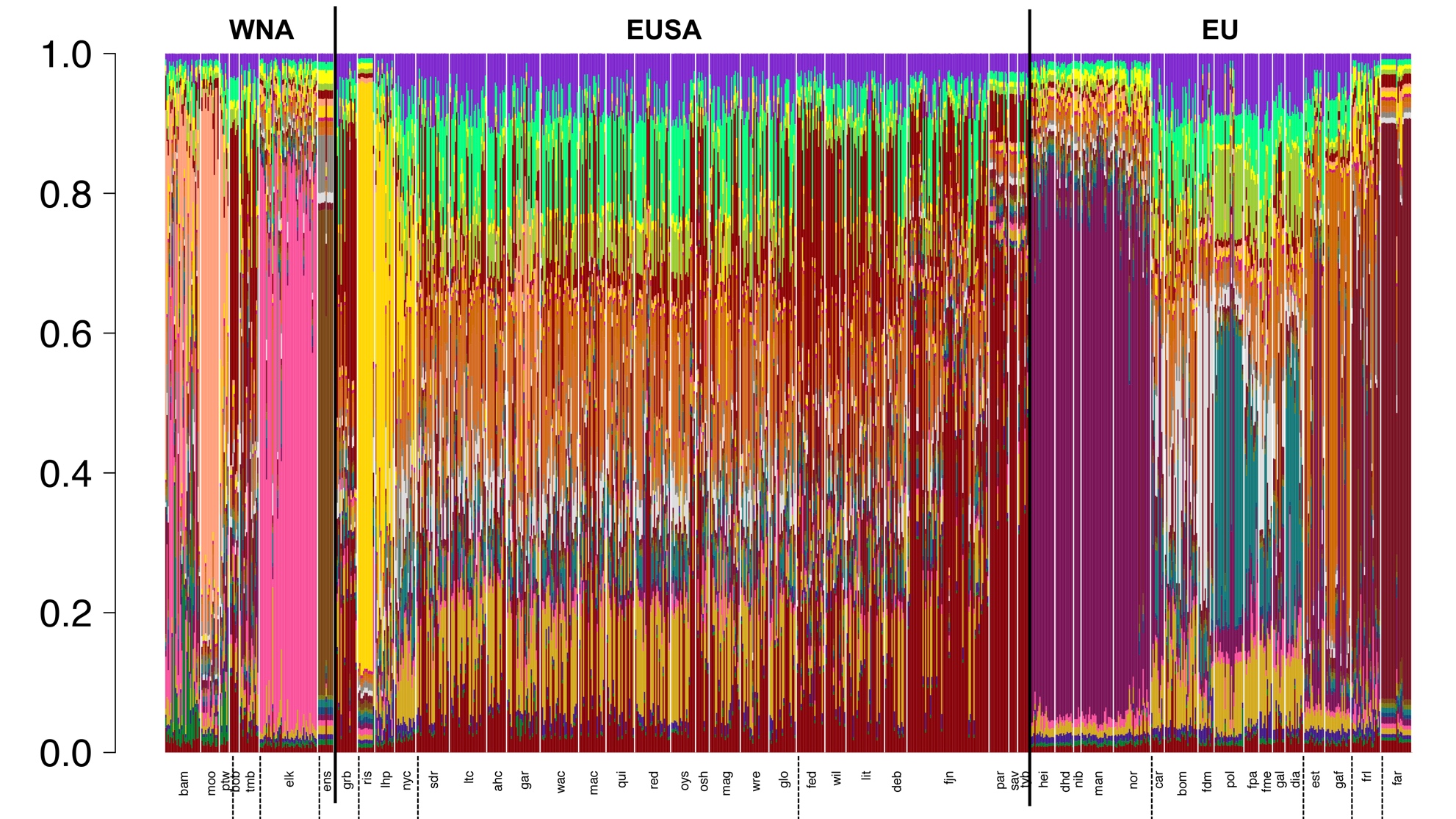


**Table S3.** The sample size of thalli and sites for the subregions used to calculate mean assignment to one of five genetic clusters identified using Bayesian clustering in *instruct* and visualized using *clumpak*.

| **Region** | **Sites** | **Number of thalli** |
| --- | --- | --- |
| Native | qin, ron (2) | 36 |
| Native | dae, gye (3) | 49 |
| Native | jin (1) | 44 |
| Native | odo (1) | 8 |
| Native | waj, tan (2) | 61 |
| Native | shk (1) | 26 |
| Native | hik, ori, shb, hir, kae, isu, ikt, jig, ikn, shn, tsu, yur, ten, yos, ftr (15) | 223 |
| Native | don, usu, shr, sar (4) | 66 |
| Native | jon (1) | 27 |
| Native | hay, fut, mou, nag (4) | 81 |
| Native | aka, mng, sou, hit (4) | 83 |
| Introduced; WNA | bam, pmo, ptw (3) | 49 |
| Introduced; WNA | bob, tmb (2) | 22 |
| Introduced; WNA | elk (1) | 45 |
| Introduced; WNA | ens (1) | 12 |
| Introduced; EUSA | grb (1) | 16 |
| Introduced; EUSA | ris, lhp, nyc (3) | 44 |
| Introduced; EUSA | sdr, ltc, ahc, gar, wac, qui, mac, red, oys, osh, mag, wre, glo (13) | 290 |
| Introduced; EUSA | fed, wil, lit, deb, fjn, par, sav, tyb (8) | 175 |
| Introduced; EU | hei, dhd, nib, man, nor (5) | 67 |
| Introduced; EU | car, bom, fdm, pol, fpa, fme, gal, est, gaf, dia (10) | 150 |
| Introduced; EU | frl (1) | 23 |
| Introduced; EU | far (1) | 24 |

**Figure S4.** Genetic differentiation of *Gracilaria vermiculopyhlla* sites along the coastlines of native Japanese and non-native WNA, EUSA and EU coastlines. Pairwise genetic distances, measured by allele identity (*F_ST_*) and allele size (*ρ_ST_*), are plotted against pairwise geographic distances (km) for each coastline. For pairwise *F_ST_*, all slopes were significantly different from zero following sequential Bonferroni correction except for slope along the EUSA (significant *p*-values are shown in bold). In contrast, for pairwise *ρ_ST_*, only the slope along the EU coastline was significantly different from zero.


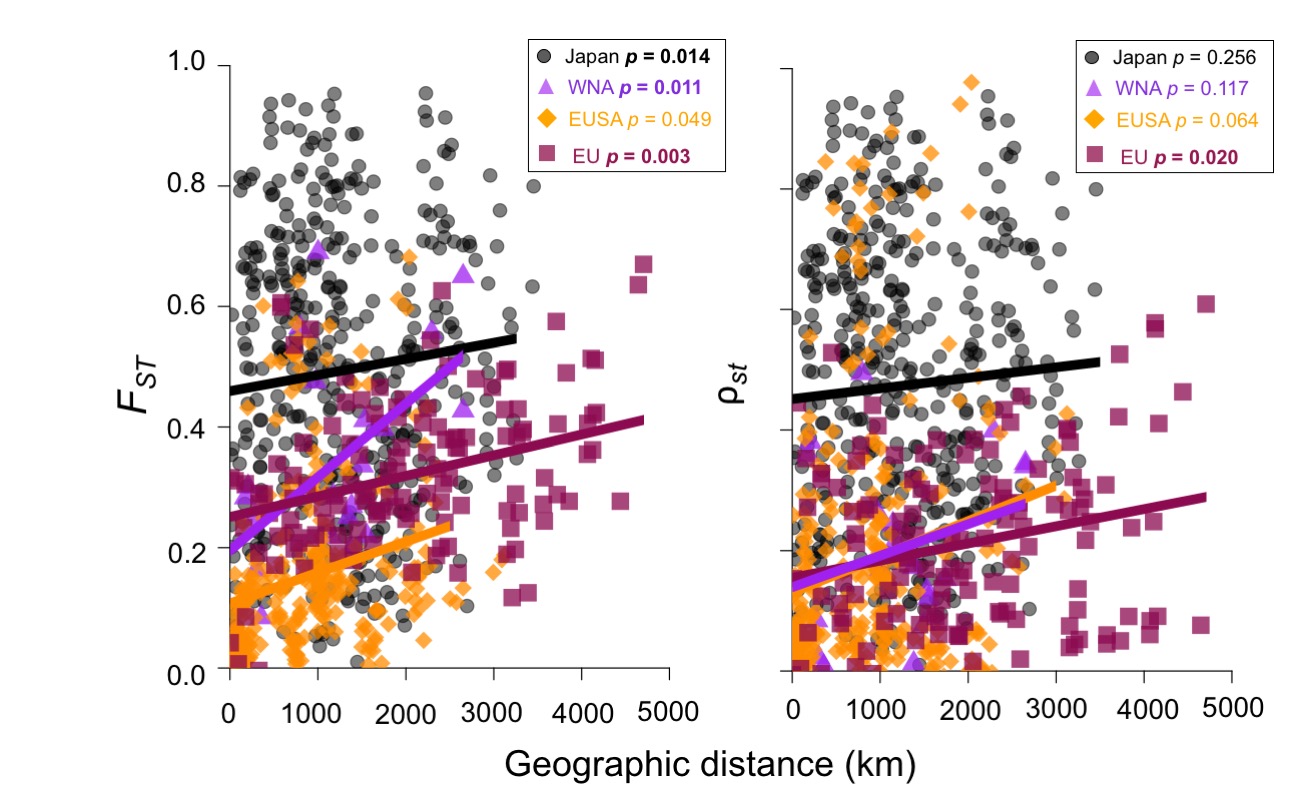


**Table S4**. Mantel’s *r*, slope, y intercept and *p-*value for Mantel tests across native and non-native coastlines for a) *F_ST_* and b) ***ρ_ST_*** as calculated using *genpop*. For allele identity, all slopes were significantly different from zero following sequential Bonferroni correction and are shown in bold except for the EUSA. For allele size, the only significant slope was the EU.

a)

| **Statistic: *F_ST_*** |  |  |  |  |
| --- | --- | --- | --- | --- |
| **Region** | **Mantel's *r*** | **slope** | **y-intercept** | ***p*** |
| Japan only | 0.153 | 2.67x10^-5^ | 0.46 | **0.014** |
| WNA | 0.544 | 1.22x10^-4^ | 0.20 | **0.011** |
| EUSA | 0.556 | 5.13x10^-5^ | 0.11 | 0.049 |
| EU | 0.365 | 3.42x10^-5^ | 0.25 | **0.003** |

b)

| **Statistic: *ρ_ST_*** |  |  |  |  |
| --- | --- | --- | --- | --- |
| **Region** | **Mantel's *r*** | **slope** | **y-intercept** | ***p*** |
| Japan only | 0.059 | 1.76x10^-5^ | 0.45 | 0.256 |
| WNA | 0.286 | 5.32x10^-5^ | 0.14 | 0.117 |
| EUSA | 0.196 | 7.28x10^-5^ | 0.13 | 0.064 |
| EU | 0.262 | 2.85x10^-5^ | 0.15 | **0.02** |

**Table S5.** ANOVA tables for each coastline and diversity metric. Significant *p*-values are shown in bold.

| Response: | *H_E_* |  |  |  |  |
| --- | --- | --- | --- | --- | --- |
|  | **df** | **SS** | **MeanSq** | **F** | **p** |
| Native lat | 1 | 0.005775 | 0.0057752 | 0.6493 | 0.426 |
| I(lat^2) | 1 | 0.005651 | 0.0056513 | 0.6354 | 0.4309 |
| Residuals | 34 | 0.302421 | 0.0088947 |  |  |
|  |  |  |  |  |  |
|  | **df** | **SS** | **MeanSq** | **F** | **p** |
| WNA lat | 1 | 0.043719 | 0.043719 | 11.812 | **0.02637** |
| I(lat^2) | 1 | 0.061765 | 0.061765 | 16.688 | **0.01504** |
| Residuals | 4 | 0.014805 | 0.003701 |  |  |
|  |  |  |  |  |  |
|  | **df** | **SS** | **MeanSq** | **F** | **p** |
| EUSA lat | 1 | 0.000605 | 0.0006049 | 0.1488 | 0.70341 |
| I(lat^2) | 1 | 0.014243 | 0.0142431 | 3.5031 | 0.07461 |
| Residuals | 22 | 0.08945 | 0.0040659 |  |  |
|  |  |  |  |  |  |
|  | **df** | **SS** | **MeanSq** | **F** | **p** |
| EU lat | 1 | 0.002318 | 0.0023182 | 0.3887 | 0.543 |
| I(lat^2) | 1 | 0.003364 | 0.0033644 | 0.5641 | 0.4651 |
| Residuals | 14 | 0.083502 | 0.0059644 |  |  |

| Response: | *A_E_* |  |  |  |  |
| --- | --- | --- | --- | --- | --- |
|  | **df** | **SS** | **MeanSq** | **F** | **p** |
| Native lat | 1 | 0.0006 | 0.00055 | 0.0027 | 0.9586 |
| I(lat^2) | 1 | 0.0936 | 0.093634 | 0.465 | 0.4999 |
| Residuals | 34 | 6.8458 | 0.201347 |  |  |
|  |  |  |  |  |  |
|  | **df** | **SS** | **MeanSq** | **F** | **p** |
| WNA lat | 1 | 0.68634 | 0.68634 | 4.943 | 0.09028 |
| I(lat^2) | 1 | 0.66534 | 0.66534 | 4.7918 | 0.09379 |
| Residuals | 4 | 0.5554 | 0.13885 |  |  |
|  |  |  |  |  |  |
|  | **df** | **SS** | **MeanSq** | **F** | **p** |
| EUSA lat | 1 | 0.07845 | 0.07845 | 2.0266 | 0.1686 |
| I(lat^2) | 1 | 0.95936 | 0.95936 | 24.7834 | **5.55E-05** |
| Residuals | 22 | 0.85161 | 0.03871 |  |  |
|  |  |  |  |  |  |
|  | **df** | **SS** | **MeanSq** | **F** | **p** |
| EU lat | 1 | 0.0871 | 0.087103 | 1.6473 | 0.2202 |
| I(lat^2) | 1 | 0.14621 | 0.146208 | 2.7651 | 0.1186 |
| Residuals | 14 | 0.74028 | 0.052877 |  |  |

| Response: | *eMLG* |  |  |  |  |
| --- | --- | --- | --- | --- | --- |
|  | **df** | **SS** | **MeanSq** | **F** | **p** |
| Native lat | 1 | 0.119 | 0.11912 | 0.0481 | 0.8277 |
| I(lat^2) | 1 | 0.535 | 0.53474 | 0.216 | 0.6451 |
| Residuals | 34 | 84.175 | 2.47573 |  |  |
|  |  |  |  |  |  |
|  | **df** | **SS** | **MeanSq** | **F** | **p** |
| WNA lat | 1 | 11.507 | 11.5066 | 1.9691 | 0.2332 |
| I(lat^2) | 1 | 18.204 | 18.2045 | 3.1153 | 0.1523 |
| Residuals | 4 | 23.374 | 5.8435 |  |  |
|  |  |  |  |  |  |
|  | **df** | **SS** | **MeanSq** | **F** | **p** |
| EUSA lat | 1 | 5.511 | 5.511 | 2.3792 | 0.1372224 |
| I(lat^2) | 1 | 40.26 | 40.26 | 17.381 | **0.0003993** |
| Residuals | 22 | 50.959 | 2.316 |  |  |
|  |  |  |  |  |  |
|  | **df** | **SS** | **MeanSq** | **F** | **p** |
| EU lat | 1 | 0.286 | 0.286 | 0.0787 | 0.7832 |
| I(lat^2) | 1 | 6.928 | 6.9277 | 1.9067 | 0.189 |
| Residuals | 14 | 50.867 | 3.6334 |  |  |

**Figure S5.** Bootstrapped (1000 replicates), unrooted neighbor-joining trees using sites with 10 or more thalli and jaccard distance for a) the native and non-native sites rooted in the native C site Qingdao (qin) and b) the non-native range alone rooted in the WNA site

Elkhorn Slough (elk). Few of the nodes in either tree had bootstrap support exceeding 0.5. The colors correspond to those used in the DAPC analyses where blue: ‘C’ haplotypes; red: ‘T’ haplotypes; purple: WNA; orange: EUSA; maroon: EU. Site codes can be found in Table S1.

a)

b)

**Figure S6.** Site assignment as inferred by DAPC using supplementary individuals as observations that did not participate in constructing the model. The supplementary individuals were predicted using a model fitted to other “training” data. The assigned sites are shown along the y-axis and the collected sites are shown along the x-axis. The probability of assignment to sites in the native range is shown with a heatmap ranging from white (0.0: no assignment) to black (1.0: complete assignment). As Eld’s Inlet had only one thallus after clones were removed, this site was removed from analyses. a) Native sites with >20% re-assignment of non-native thalli are shown in larger text and bold (**, mng had ~40% of non-native thalli assigned to it). Native sites with 1% < x < 5% reassignment of non-native thalli are shown in smaller text. Native sites with < 1% re-assignment are not shown on the map. b) WNA, EUSA and EU were each projected separately onto discriminant functions obtained from the native range the other two non-native coastlines. For example, the WNA sites were excluded from model construction. Then, each of the WNA sites were projected on the discriminant functions constructed from the native range, EUSA and EU (hence, the “x” filling the cells along the assigned WNA sites as they could not be assigned to their own site or subregion). The probability of assignment is shown with a heatmap ranging from white (0.00: no assignment) to black (1.00: complete assignment). As Eld’s Inlet had only one thallus after clones were removed, this site was removed from analyses.

a)

b)


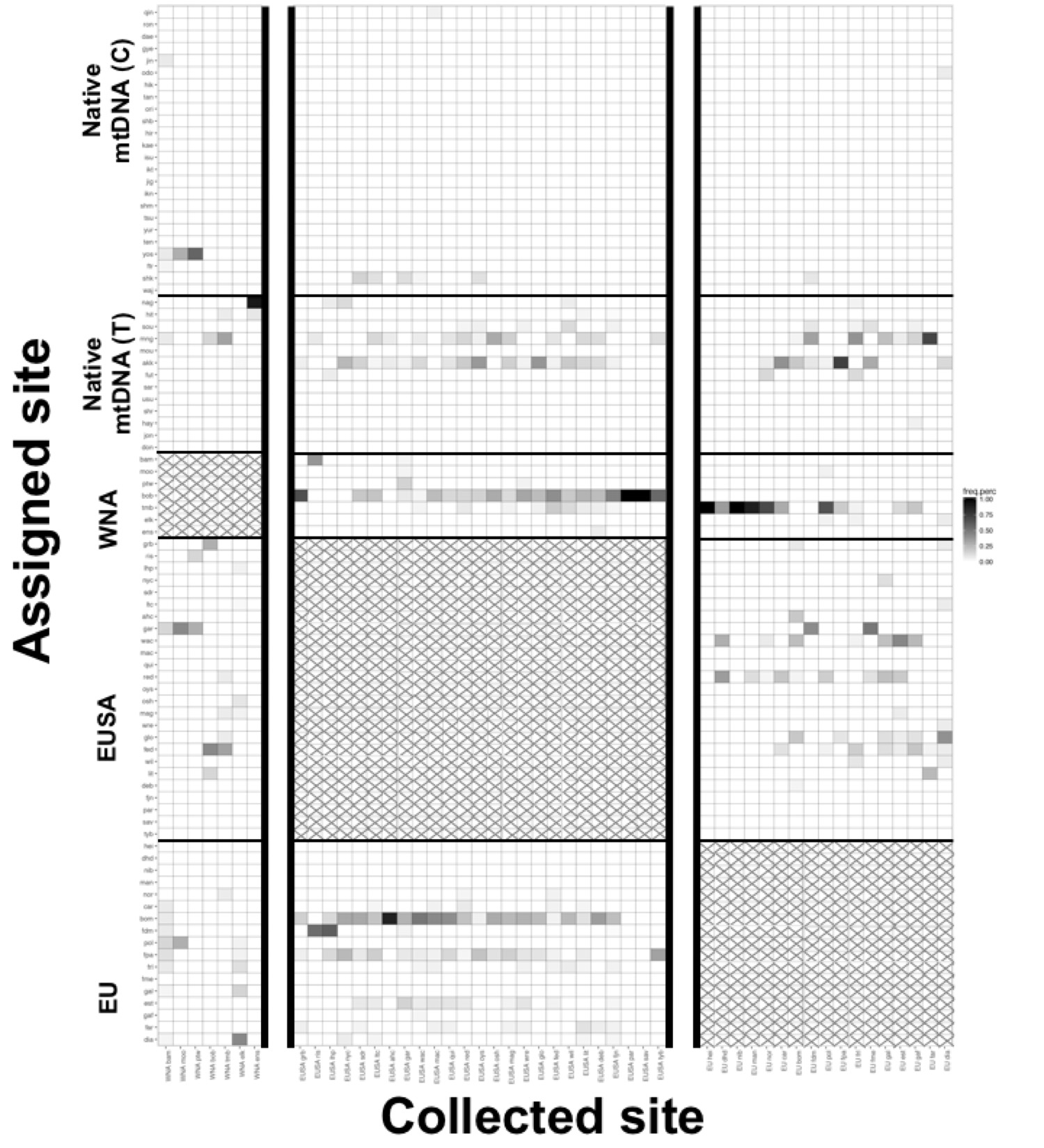

Supplement: Supplementary file 1 [file ECE3-7-4432-s001.docx]
